# Smad2 Cooperating with TGIF2 Contributes to EMT and Cancer Stem Cells Properties in Pancreatic Cancer via Co-Targeting SOX2

**DOI:** 10.7150/ijbs.102381

**Published:** 2025-01-01

**Authors:** Fuqiang Zu, ChuanPing Chen, Qilong Geng, Haoyu Li, Boyuan Chan, Guopei Luo, Mengcheng Wu, Matthias Ilmer, Bernhard W Renz, Lutterodt Bentum-Ennin, Hao Gu, Weiwei Sheng

**Affiliations:** 1 Department of General Surgery, the Second Affiliated Hospital of Anhui Medical University, Hefei, 230601, China.; 2 Department of Pharmacy, The First Affiliated Hospital of Anhui Medical University, Hefei, Anhui 230022, China.; 3 Department of Clinical Medicine, Anhui Medical University, Hefei, 230022, China.; 4 Department of Pancreatic Surgery, Fudan University Shanghai Cancer Center, Shanghai, 200032, China.; 5 Department of General Surgery, the First Affiliated Hospital of Anhui Medical University, Hefei, 230022, China.; 6 German Cancer Consortium (DKTK), Partner Site Munich and German Cancer Research Center (DKFZ), Heidelberg, Germany.; 7 Department of Immunology, School of Basic Medical Sciences, Anhui Medical University, Hefei 230032, China.

**Keywords:** TGIF2, SOX2, EMT, cancer stem cells, pancreatic cancer

## Abstract

The underlying mechanisms between cancer stem cells (CSC) and epithelial-mesenchymal transition (EMT) in pancreatic cancer (PC) remain unclear. In this study, we identified TGIF2 as a target gene of CSC using sncRNA and machine learning. TGIF2 is closely related to the expression of SOX2, EGFR, and E-cadherin, indicating poor prognosis. Mechanistically, TGIF2 promoted the EMT phenotype and CSC properties following the activation of SOX2, Slug, CD44, and ERGF/MAPK signaling, which were rescued by SOX2 silencing. TGIF2 silencing contributes to the opposite phenotype via SOX2. Notably, Smad2 cooperates with TGIF2 to co-regulate the SOX2 promoter, which in turn promotes EMT and CSC signaling by transactivating Slug and EGFR, respectively. The transactivation of EGFR/MAPK signaling by SOX2 promotes TGIF2 nuclear translocation, forming a positive feedback loop *in vitro*. Moreover, the interaction of TGIF2 and SOX2 with EGFR inhibitors promoted subcutaneous tumors and liver metastasis *in vivo*. Thus, the TGIF2/SOX2 axis contributes to CSC, EMT, and chemoresistance, providing a promising target for PC therapy.

## Introduction

Pancreatic cancer (PC) is a lethal tumor with approximately 11% surviving over 5 years 1, 2 owing to drug resistance, local progression, and distant metastasis. Cancer stem cells (CSC) and epithelial-mesenchymal transition (EMT) play significant roles in tumor spread and distant metastasis 3. CSC act as a self-renewing pool of tumorigenic cells, which can lead to drug resistance, recurrence, metastasis, and aggressiveness of cancer 4, 5. The active tumorigenesis of CSC also paves the way for EMT, which has been demonstrated in PC chemoresistance and metastasis 6-8. For example, zinc finger E-box binding homeobox 1 (ZEB1) positively regulates CD44 to regulate CSC properties 9. Our previous studies identified several critical oncogenes as potential EMT promoters in CSC 10-13. It is essential to elaborate on the molecular interconnection between CSC and EMT and their regulation.

Previous Single-cell RNA (scRNA) analyses have revealed the composition and interactions of CSC in the tumor microenvironment. Highly expressed genes associated with CSC have been identified that interact with EMT, oxidative stress, proteasomes, and immunotherapy 14-16. However, comprehensive crosstalk between CSC signatures and the EMT landscape remains limited 17, 18. Here, we investigated the cellular composition and transcriptome profiles of CSC using sncRNAs and machine learning 19. A novel machine learning framework that incorporates 10 algorithms was established, composed of six genes, and indicated excellent prognosis prediction. Among these, TGIF2 has been identified as the most significant factor at the transcriptional intersection of CSC and EMT. Therefore, we explored its role in maintaining CSC pluripotency and promoting EMT in PC.

TGF-β-induced factor homeobox (TGIF) is a family of transcription factors, containing multiple members, such as TGIF1, TGIF2, TGIF2LX, and TGIF2LY 20, 21. As a DNA-binding transcription factor, TGIF2 is important for regulating many crucial developmental processes, including cell proliferation and differentiation, and is involved in the development of several cancers, including melanoma, osteosarcoma, oral squamous cell carcinoma, glioblastomas, and lung cancer 22-25. However, its potential role in PC has not been studied. In this study, the TGIF2/SOX2 transcriptional axis promoted PC development by facilitating EMT and maintaining CSC pluripotency. This study identified a novel transcriptional regulatory network for revealing the molecular mechanisms underlying the malignant biology of PC.

## Materials and methods

### Patients and samples

All three surgical resection samples diagnosed with PC were collected at the First Affiliated Hospital of Anhui Medical University. Briefly, the samples were chopped on ice, dissociated in a collagenase digestion solution, and oscillated at 50 rpm. The cells were filtered using a strainer and the survival rate was measured. For Single-cell RNA sequencing (scRNA), the single-cell suspension was adjusted to 1×105 cells/mL and sequenced by 10× Genomics on an Illumina NovaSeq instrument with 150bp paired-end reads. Raw sequencing data were converted into FASTQ files using Illumina bcl2fastq, and aligned to the human genome reference sequence (GRCH38). The SeekSoul® Tools was used to sample demultiplexing, barcode processing, and gene counting to generate a digital gene-cell matrix. The gene expression matrix was processed and analyzed using the Seurat package (version 5.0.3) 26.

### ScRNA and bulk analysis

Seurat was used for dimensionality reduction, clustering, and visualization 27. Filtered gene expression was examined at 300-10000, and the mitochondrial percentage was set at lower than 20. Data were normalized and identified for variable genes, clustered with a resolution of 0.5, and visualized using t-SNE. Cluster markers were identified using FindAllMarkers and were re-annotated for further analysis.

The transcriptional data and clinical information were downloaded from four public databases: E-MTAB-6134, TCGA, GSE21501 and GSE62452 28, 29. Further, 335 stem genes were identified using the StemChecker 30. Briefly, the PC stemness scores of PC was estimated using single-sample gene set enrichment analysis (ssGSEA) via GSVA packages (V 1.50.1) 31 and categorized into CSC_high and CSC_low groups. For machine learning, we validated the CSC index of PC prognosis in the E-MTAB-6134 database (training cohort) and TCGA database (validation cohort) and selected the best model of the machine learning algorithms 32. The gene coefficients of the model were calculated by multivariate Cox regression analysis, and the final CSC-index = Σ (Coefi × Exp) was obtained. We compared the OS of the groups via Kaplan-Meier analysis, evaluated the predictive performance through receiver operating characteristic (ROC) curve analysis, and validated independent prognostic factors by univariate and multivariate Cox regression analysis using survival packages (V 3.5-8).

Functional analysis was performed using clusterProfiler (V 4.10.1) and org. Hs. eg. db packages (V 3.18.0) using GSEA analysis 33. Differences in the enriched HALLMARK pathways were compared between patients with high and low CSC-index.

Target genes of TGIF2 were predicted using the TFDB 3.0 database 34. Common genes were identified and the score was calculated using TGIF2. ATAC-seq data were downloaded from the GSE213394 database 35. The chromatin openness of these genes was also determined. Moreover, we downloaded EMT genes from the MSigDB database and evaluated the EMT signatures of PC and Gastric cancer (GC) from previous studies 36-38.

### Tissue specimens and PC cell lines

Here, 88 cases of paraffin-embedded PC specimens and 56 adjacent pancreatic specimens were collected for the IHC assays. Twenty patients with PC and their adjacent tissues were randomly selected and used for PCR assays. All samples were obtained from patients who underwent radical surgery without neoadjuvant therapy, and the diagnosis was confirmed by a pathologist. All patients provided written informed consent and the study was approved by the Ethics Committee of the Second Affiliated Hospital of Anhui Medical University (2023558). Cells were cultured as described previously 10-13. Five cell lines (AsPC-1, BxPC-3, PANC-1, Miapaca-2, and HEK-293) were purchased from the National Cell Culture in Shanghai using recommended growth media containing 10% fetal calf serum (Hyclone, Logan, UT, USA) 10.

### Immunohistochemistry assays

Immunohistochemistry was performed as previously described 10-13. The following primary antibodies were used and incubated overnight: anti-TGIF2 (Proteintech, Chicago, IL, USA, Cat #11522-1-AP, dilution 1:100), SOX2 (Proteintech, Cat #66411-1-Ig, dilution 1:100), EGFR (Proteintech, Cat #66455-1-Ig, 1:500 dilution), and anti-E-cad (Abcam, Cambridge, UK, Cat #ab40772, dilution 1:200).

### Western blot analysis

Western blot (WB) was performed as previously described 10-13. Nucleoplasmic proteins were extracted from the cells using a special lysis buffer (BB-36021, BestBio) following the manufacturer's instructions. The antibodies used were TGIF2 (Proteintech, dilution at 1:500), SOX2 (Proteintech, dilution at 1:1000), E-cad (Abcam, dilution at 1:1000), N-cadherin (N-cad, Proteintech, Cat # 22018-1-AP, dilution at 1:1000), Vimentin (Proteintech, Cat # 10366-1-AP, dilution at 1:2000), Snail1 (Proteintech, Cat #13099-1-AP, dilution at 1:500), Slug (Proteintech, Cat #12129-1-AP dilution at 1:500), CD133 (Proteintech, Cat #18470-1-AP, dilution at 1:1000), CD44 (Proteintech, Cat #15675-1-AP, dilution at 1:1000), EGFR (Proteintech, dilution at 1:1000), p-EGFR (Abcam, Cat # ab97613, dilution at 1:500), p-ERK (Cell Signaling Technology, Beverly, USA, Cat #137F5, dilution at 1:1000), Smad2 (Cell Signaling Technology, Cat #5339, dilution at 1:1000), and GAPDH (Proteintech, Cat #60004-1-Ig, 1:3000).

### Co-Immunoprecipitation (Co-IP)

Based on our previous study 10-13, the lysate of BxPC-3 was extracted using RIPA lysis buffer. TGIF2 antibodies mixed with magnetic beads were incubated together on a rotator overnight in 4°C. The final immunocomplex was stripped by boiling in WB loading buffer. The input and IgG panels were used in parallel as positive and negative controls, respectively.

### qRT-PCR

qRT-PCR was performed as described previously 10-12. mRNA levels in tissues and cell lines were estimated using a LightCycler kit for the fast qRT-PCR system. The primers used are listed in Table S1. The quality of PCR products was monitored using post-PCR melt-curve analysis. The expression level of these target genes was calculated by the -△△Ct method.

### siRNA and lentivirus vector-mediated TGIF2/SOX2 overexpression

The siRNA and shRNA sequences for TGIF2 and SOX2 were summarized in Table S2, which were synthesized by GenePharma Co., Ltd. (Shanghai, China). shRNA-mediated TGIF2 and SOX2 silencing and lentivirus vector (GV492 and CV186)-mediated TGIF2, SOX2, and Smad2 overexpression (TGIF2-OE, SOX2-OE, and Smad2-OE, respectively) were purchased from GeneChem (Shanghai, China). siRNAs and plasmids were mixed with Oligofectamine 3000 (Invitrogen, Carlsbad, CA, USA) according to the manufacturer's instructions.

EMT model construction

EMT model construction has been described in our previous studies 10-13. Briefly, cells were induced by TGFβ and calculated the percentage of residual epithelial cells to the whole cell area. We validated EMT phenotypes by western blotting and detected cell mobility in EMT-stimulated cells using invasion and migration assays.

### MTT and migration assays

Based on our previous studies 10-13, MTT assay was used to detect cell proliferation and gemcitabine resistance. Briefly, transfected cells were incubated for growth with or without Erlotinib treatment (5 µM for 2 h twice), different concentrations of Gemcitabine, respectively. Then, we treated the cells with MTT and DMSO successively and measured the absorbance at 570 nm using an ELISA microtiter plate reader (Bio-Rad 680, California, USA). For the migration assays, transfected cells were implanted into the upper chamber with Matrigel or serum-free medium. Medium supplemented with 10% FBS was added to the bottom as a stimulus. The migrated cells were fixed, co-stained with crystal violet, and counted in five random fields 10.

### CSC culture

According to previous studies 39, transfected PANC-1 and BxPC-3 cell lines were cultured in serum-free DMEM-F12 medium containing 0.4% BSA (Sigma), N-2 Plus media (Gibco), B-27 (Gibco), FGF (10ng/ml) and EGF (20ng/ml, for Kras wildtype BxPC-3 cells only) (Preprotech, Rocky Hill, NJ, USA) at a density of 15000 cells/ml in low-attachment dishes (Corning, NY, USA). Round aggregates containing six or more cells were considered as 'spheres' for quantification purposes. The secondary spheres formed following a 1-2-week incubation were counted and photographed.

### Organoids culture from pancreatic acinar cells

Wild-type C57BL/6 mice and Pdxcre; LSL-KrasG12D (KC) mice were a gift from Timothy Wang's laboratory at Columbia University and were kept in the animal experimental department of Anhui Medical University under SPF conditions. The pancreatic tissue was rinsed in cold PBS and chopped into pieces, mixed with collagenase and stirred at 37°C for 20 min. The cells were resuspended in PBS, incubated in Pancreatic Medium, mixed with Matrigel, and seeded in a 24 well plate for 3-5 days. Once the organoids were formed, the whole organoids released from the 3D Matrigel were transfected with TGIF2 and SOX2 siRNAs to achieve the highest transfection efficiency, according to protocols described in a previous study 40. After transfection, the organoids were cultured for another seven days, counted, and photographed.

### Immunofluorescence (IF) staining

As detailed in our previous studies 10-13, transfected cells were implanted with or without erlotinib treatment, fixed in paraformaldehyde, permeabilized with Triton, incubated with BSA, and stained with primary antibodies against TGIF2 and SOX2 overnight. The plates were then incubated with secondary antibodies of different origins (rabbit FITC for TGIF2 and mouse TRITC for SOX2). Hoechest33258 was used for nuclear visualization. IF was performed in triplicates.

### Chromatin immunoprecipitation (Chip) assay

The chip assay was performed according to manufacturer's instructions (9003; Cell Signaling Technology). Briefly, cultured cells were lysed in protease inhibitor buffer and sonicated to extract approximately 150-800bp chromatin fragments. Following dilution with an IP dilution buffer, the lysates were incubated at 4°C overnight with TGIF2, Smad2 or SOX2 antibodies. The antibody-bound chromatin complex was precipitated using protein A/G magnetic beads and salmon sperm DNA for 4 h at room temperature. Finally, DNA was isolated from the immunoprecipitated chromatin using a DNA Elution Buffer. The corresponding PCR-amplified primer pairs flanking the consensus-binding sites in the SOX2, Slug, and EGFR promoters are shown in Table S1. All the PCR was carried out for 35 cycles with the primers annealed at 58°C, and the PCR products were resolved on a 2% agarose gel in TBE buffer for the final gel imaging (Bio-Rad).

### Dual-luciferase reporter assay

SOX2-OE transfected HEK-293 cells were seeded in 24-well plates and co-transfected with a pGL3-Basic-SOX2 promoter plasmid (SOX2-WT), the corresponding mutant plasmid (SOX2-Mutant, SOX2-MT) for TGIF2, and the corresponding mutant plasmid (SOX2-Mutant2, SOX2-MT2) for Smad2. For the dual-luciferase assay, wild-type (wt) and mutant (mut) EGFR promoter plasmids were constructed. HEK293 cells in SOX2-OE and scramble groups were seeded and co-transfected with 0.5-1 μg of EGFR-WT and EGFR-Mut promoter plasmid and empty vector using Lipofectamine 3000. After incubation for 48 h, the luciferase activity was measured using the Dual-Luciferase® Assay Kit (Cat. #E2920, Promega). The results were repeated at least three times.

### *In vivo* xenograft model

All animal experiments were approved by the Animal Care Committee of Anhui Medical University (LISC20231510). Twelve nude mice were randomly divided into four groups: scramble, TGIF2-OE, TGIF2-OE/sh-SOX2, and TGIF2-OE plus erlotinib, and injected into the axillae using transfected PANC-1 cells. According to a previous study 12, the TGIF2-OE plus erlotinib group was orally administered 100 mg/kg/day erlotinib for 5 days per week, whereas the other groups were orally administered 1% DMSO as a control. All mice were sacrificed after 3 weeks. Tumor volumes were calculated using the following formula: length × width × height × 0.5 in mm3. Moreover, BxPC-3 cells were injected into the spleens of 15 nude mice to construct a distant liver metastasis model (n=5 in each group), which was assessed by the number of liver metastases and liver weight/body weight ratio 41, 42. All the mice were euthanized 4 weeks later. Tissue samples were extracted and used for hematoxylin, eosin (HE) and IHC staining.

### Statistical analysis

All statistical analyses were performed using the SPSS software (version 21.0; R Software 4.3.1). Significant differences were analyzed using Welch's t-test for two-group comparisons, one-way ANOVA for multiple group comparisons, two-way ANOVA with Tukey's test or Sidak's test for interactions, log-rank test for survival data, Pearson's correlation analysis, chi-square test, and Kruskal-Wallis test for nonparametric data analysis 43. Statistical significance was set at P <0.05 was considered significant.

## Results

### Identification of TGIF2 as a targeted gene of CSC and EMT

**Figure 1:**
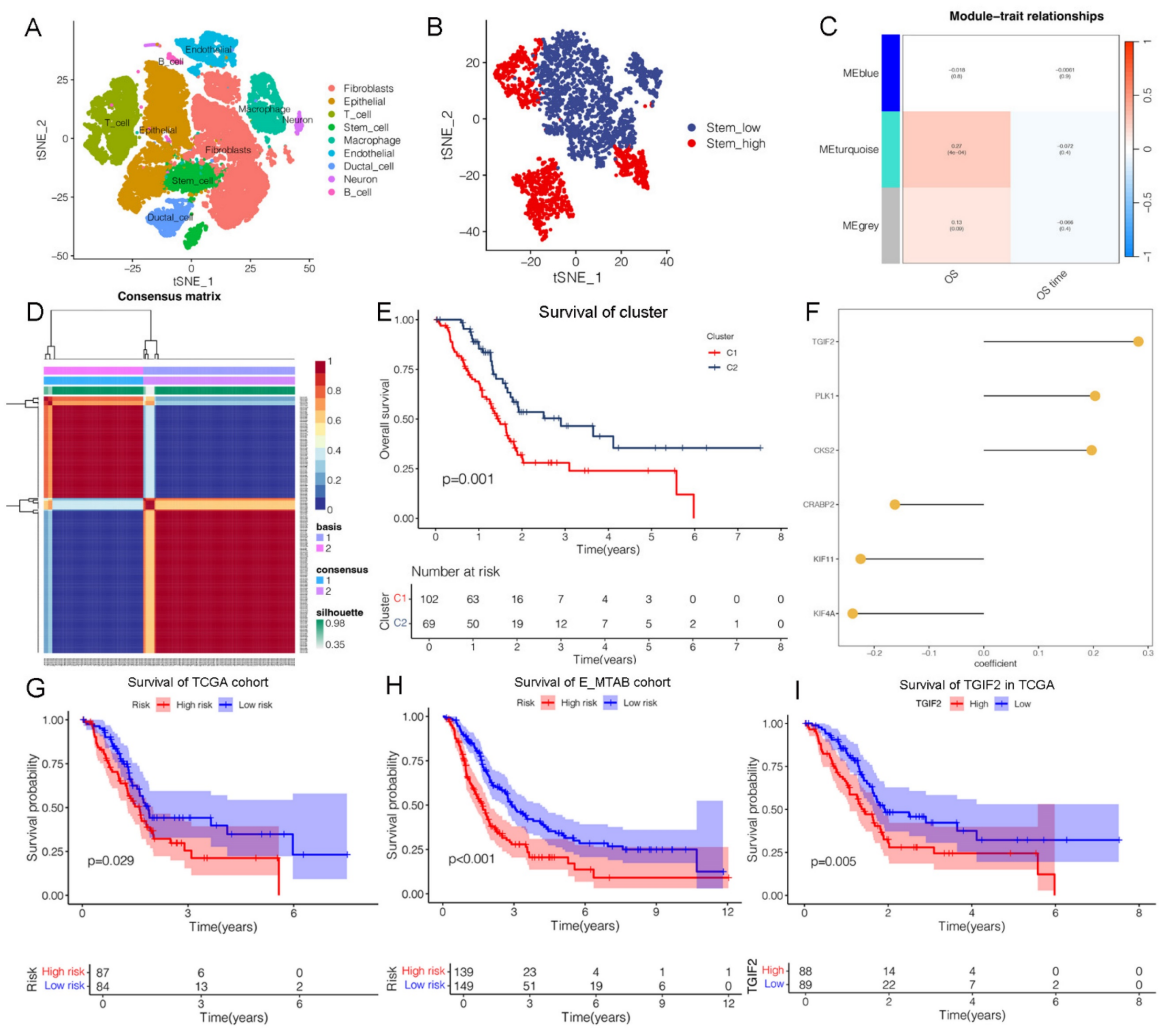
Identification of TGIF2 as a CSC gene. **A.** Nine clusters of tSNE distribution in scRNA analysis. **B.** Cell_type of tSNE plotter in stem subcluster. **C.** Correlation map of 3 modules with OS time and status. **D.** the Optimal grouping of risk scores via NFM algorithm. **E.** Survival difference between stem subgroups. **F.** The qualified CSC genes in risk model. **G and H.** The survival difference between the high and low-risk in TCGA cohort and E-MTAB cohort. **I.** The overall survival of TGIF2 in TCGA database by Kaplan Meier database.

Based on marker annotation, we preliminarily identified nine cell subtypes, including fibroblasts, epithelial cells, T cells, stem cells, macrophages, endothelial cells, ductal cells, neurons, and B cells (Figure 1A, Figure S1A). Stem cells were subset and re-clustered into stem_high and stem_low groups using ssGSEA (Figure 1B). In total, 335 stem genes were identified and selected for further analysis (Figure S1E). Three modules were identified in the stemness signature using the WGCNA network (Figure 1C). The turquoise module showed the strongest positive correlation with OS (ME = 0.27, P = 4e-4), from which the genes were validated for further analyses (Figure S1E). We then calculated the risk scores of the genes and divided them into two categories (Figure 1D, Figure S1C-D), from which 574 subtype genes were identified (Figure S1E). Patients with subtype C2 had a significantly longer OS than those with subtype C1 (Figure 1E), which was well distinguished in the PCA plot. Thirty CSC target genes were identified (Figure S1E). The heatmap shows significant differences in the expression levels of the subtypes (Figure S1H).

We developed a machine learning model and calculated the C-index of 45 predictive models (Figure S2A). The two algorithms with the highest C-indices were the EMTAB and TCGA databases. Notably, six genes were identified in the CSC model (Figure 1F), including TGIF2, PLK1, CKS2, CRABP2, KIF11, and KIF4A, and were validated using the E-MTAB and TCGA databases (Figure S2B-G). Our findings revealed a valuable AUC for predicting the 1-, 3-, and 5-year survival rates of patients in both the E-MTAB and TCGA databases (Figure S2B-C). The CSC model showed significance only in the E-MTAB cohort (Figure S2D) compared to the TCGA cohort (Figure S2E). Additionally, a prognostic nomogram indicated good prediction of 1-, 3-, and 5-year overall survival (Figure S2F-G). Calibration curves confirmed that the nomogram outperformed the other predictors. A significant difference in survival was observed between the high- and low-risk groups (Figure 1G-H).

There exist enriched pathways of E-MTAB cohort (Figure S3A), including TGF-β, mTORC1 and Kras signaling, which are closely related to MAPK activating. An interaction between CSC and EMT was predicted, which is consistent with our hypothesis. Additionally, we validated that the CSC model was enriched in KRAS and EMT signaling in TCGA cohort (Figure S3B-C). A significant difference in survival was observed between the high- and low-risk groups based on KRAS signaling and EMT (Figure S3D-G). Correlation analysis revealed that TGIF2 expression was positively associated with Snail2 (Slug) and EGFR expression in PC tissues (Figure S3H). Therefore, we identified TGIF2 as having the highest expression, indicating poor survival (Figure 1I), and selected it for further analysis.

TGFI2 is identified as a transcript factor previously 24, 25. Based on the TFDB database, eight stem cell genes were identified as target genes (Figure S4A and Table S4), including MYC, SOX2, TP53, LMNB1, YY1, OTX2, CBX3, and HDAC1, but not OCT4 or NANOG. Next, we evaluated the correlation between TGIF2 and these genes (Figure S4D-I). SOX2 expression was strongly correlated with TGIF2 expression (r=0.3, P<0.05; Figure S4E). Moreover, SMAD3 binds to TGIF2 to increase SOX levels by forming a transcriptional complex 44, which further indicates a specific interaction between TGIF2 and SOX2.

Consistency exists between PC and GC in their EMT response. We identified 11 genes and compared their EMT signatures between PC and GC. The EMT signatures of five genes were both changed (RUNX2, SNAI2, ZEB1, TEAD1, and NUAK1), while the others were not, which indicated consistency between PC and GC in the EMT response (Figure S4B & C).

### Overexpression of TGIF2 and SOX2 indicated advanced clinical stage and dismal prognosis

**Figure 2:**
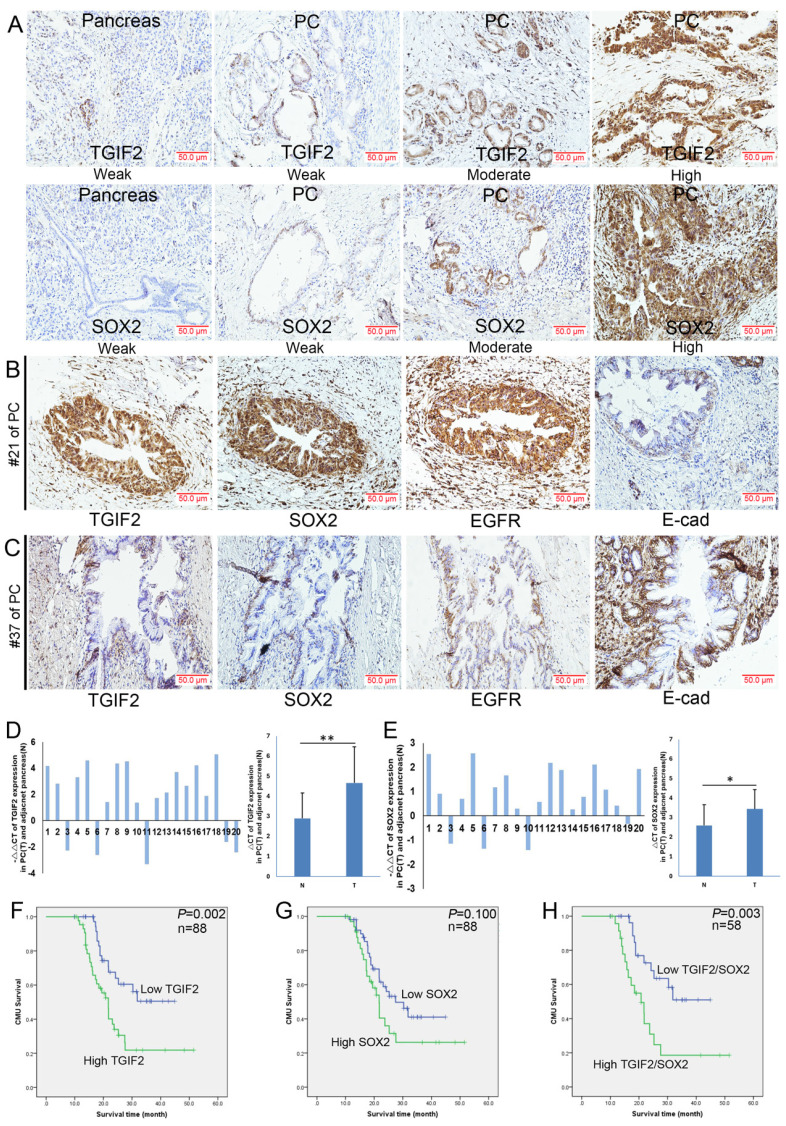
The expression of TGIF2 and SOX2 in human PC and adjacent pancreas. **A.** TGIF2 and SOX2 expression in PC and paired pancreas specimens by IHC. **B and C.** TGIF2, SOX2, EGFR and E-cad expression in two PC specimens (#21 and #37). **D.** The mRNA level of TGIF2 in 20 cases of human PC and adjacent pancreas by qRT-PCR (T: PC; N: paired pancreas) and SOX2.** E.** The mRNA level of SOX2 in our cohort. **F and G.** High and low expression of TGIF2 and SOX2 against prognosis. **H.** Combination of TGIF2 and SOX2 against prognosis.

SOX2, EGFR, and E-cadherin are significant regulators of CSCs and EMT 45, 46. In this study, both TGIF2 and SOX2 were localized in the cytoplasm and nucleus (Figure 2A), whereas EGFR showed membrane and cytoplasmic expression (Figure 2B). According to a previous study 12, an abnormal expression pattern can be classified as absent, cytoplasmic, or heterogeneous (Figure 2B). As shown in Figure 2A, the expression levels of TGIF2 and SOX2 were much higher in PC tissues than in healthy tissues (51.1% vs. 23.2%, P<0.01; and 39.8% vs. 3.6%, respectively). P<0.001). Correlation analysis showed that TGIF2 was positively associated with SOX2 and EGFR expression but negatively associated with E-cadherin expression (Table 1). High expression of TGIF2, SOX2, EGFR, and E-cadherin was observed in #21 PC specimen, #37 (Figure 2B-C).

Similarly, high levels of TGIF2 and SOX2 were also observed in randomly selected 20 cases of PC tissue samples (Table S3) compared with those in the adjacent pancreas, which was further verified according to the GEPIA database (Figure 2D-E and Figure S1F-G). The relationships between TGIF2, SOX2, and clinical traits are shown in Table 2-3. Briefly, TGIF2 overexpression was closely associated with T stage (P=0.011), lymph node metastasis (P=0.032), and TNM stage (P=0.028), whereas SOX2 overexpression was associated with tumor size (P=0.029) and T stage (P=0.026). High TGIF2 expression was associated with poor OS (P=0.002) (Figure 2F), whereas SOX2 expression was not significantly associated (Figure 2G). The combined expression of TGIF2 and SOX2 contributed to worse prognosis (Figure 2H).

### Interaction of TGIF2/SOX2 promotes EMT of PC

**Figure 3:**
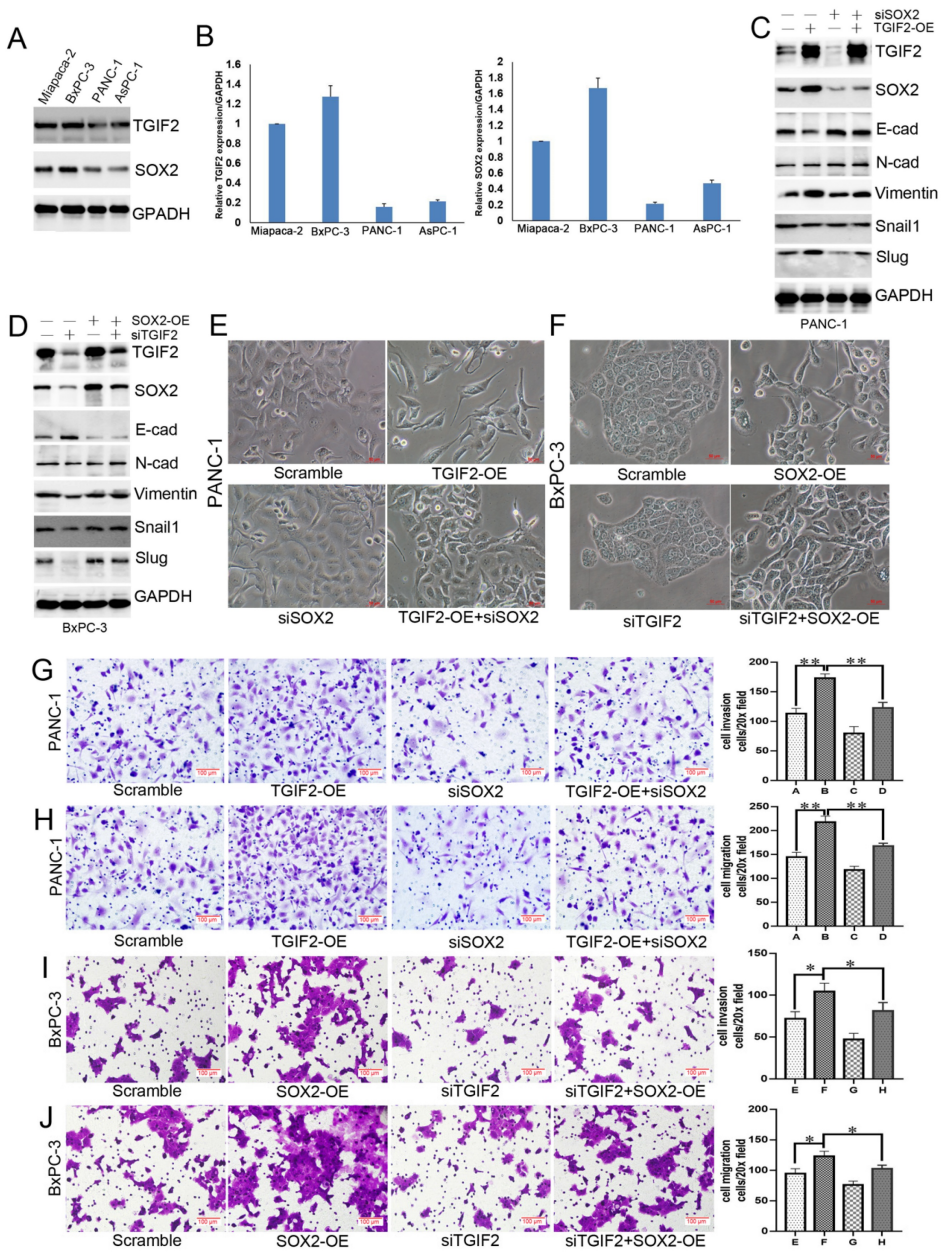
Coordination of TGIF2/SOX2 promotes EMT of PC *in vitro*. **A and B.** TGIF2 and SOX2 protein (A) and mRNA (B) level in 4 PC cell lines. **C.** The protein expression of TGIF2, SOX2 and EMT signaling in Scramble, TGIF2-OE, siSOX2 and TGIF2-OE/siSOX2 groups in PANC-1 cells. **D.** The protein expression of TGIF2, SOX2 and EMT signaling in Scramble, siTGIF2, SOX2-OE, and siTGIF2/SOX2-OE groups in BxPC-3 cells. **E.** The EMT phenotype in Scramble, TGIF2-OE, siSOX2 and TGIF2-OE/siSOX2 groups in PANC-1 cells.** F.** The EMT phenotype in Scramble, SOX2-OE, siTGIF2 and siTGIF2/SOX2-OE groups in BxPC-3 cells.** G and H.** Cell invasion (G) and migration (H) in Scramble, TGIF2-OE, siSOX2 and TGIF2-OE/siSOX2 groups in PANC-1 cells. **I and J.** Cell invasion (I) and migration (J) in Scramble, siTGIF2, SOX2-OE and siTGIF2/SOX2-OE groups Scramble, SOX2-OE, siTGIF2 and siTGIF2/SOX2-OE in BxPC-3 cells. Bars indicate ± S.E.*, P <0.05; **, P <0.01 compared with the control.

WB and qRT-PCR showed that TGIF2 and SOX2 were more highly expressed in Miapaca-2 and BxPC-3 cells than in other cells (Figure 3A-B, Figure S5A). PANC-1 and BxPC-3 cells were used for TGIF2 overexpression (TGIF2-OE) and silencing (si TGIF2) separately. TGIF2-OE led to the activation of Vimentin and Slug and the inactivation of E-cadherin and SOX2 expression. SOX2 silencing partially downregulates N-Cadherin, Vimentin and Slug expression and upregulates E-cadherin expression. Moreover, the loss of E-cadherin expression caused by TGIF2-OE was rescued by silencing SOX2 (Figure 3C, Figure S5B). Conversely, EMT signaling blunted by silencing TGIF2 was rescued by SOX2 overexpression (SOX2-OE) (Figure 3D, Figure S5C), suggesting an interaction effect of TGIF2/SOX2 on EMT signaling.

Therefore, it is essential to induce cell transfer to maintain EMT morphology. TGIF2-OE drove EMT by losing epithelial characteristics and presenting a spindle-shaped morphology (Figure 3E). Although SOX2 silencing did not affect the cell morphology, it reversed the TGIF2-OE EMT phenotype and decreased the spindle-shaped phenotype. Conversely, TGIF2 silencing inhibited the SOX2-OE phenotype in BxPC-3 cells (Figure 3F). Moreover, TGIF2 overexpression promotes the invasion and migration of PANC-1 cells. Although SOX2 silencing partially inhibited cell mobility, this inhibition was reversed in the TGIF2-OE + siSOX2 group (Figure 3G-H). In contrast, TGIF2 silencing inhibits the invasion and migration of BxPC-3 cells. However, SOX2-OE rescued the invasive and migratory capabilities of BxPC-3 cells (Figure 3I-J). These findings demonstrate the coordination of TGIF2/SOX2 in EMT.

### TGIF2/SOX2 cooperatively promote CSCs and drug resistance

**Figure 4:**
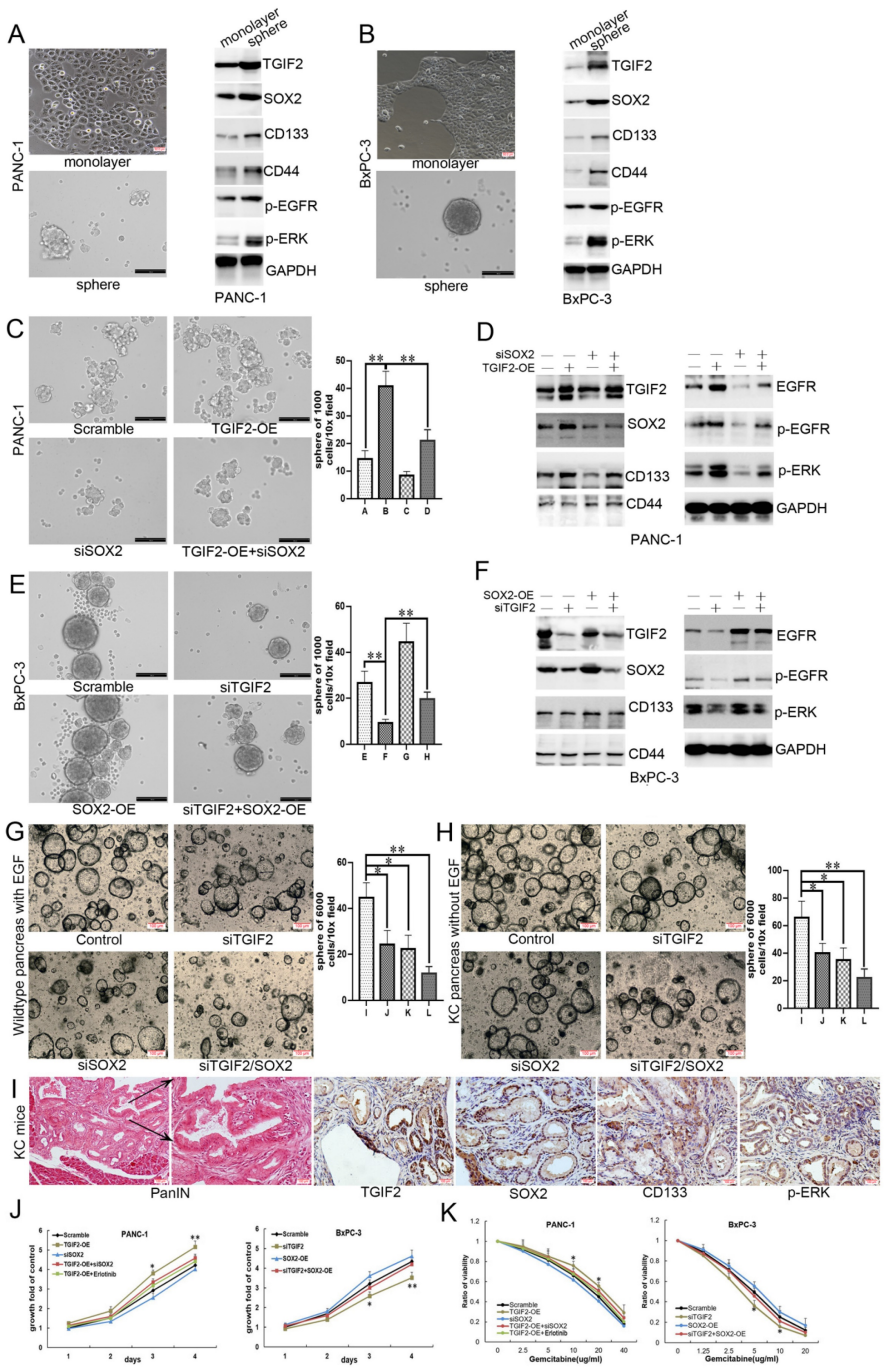
Coordination of TGIF2/SOX2 promotes CSCs and drug resistance of PC *in vitro*. **A and B.** Sphere formation in PANC-1 cells (A) and BxPC-3 (B) cells following the activation of TGIF2, SOX2, CD133, CD44 and EGFR/MAPK signaling. **C and D.** The sphere number (C) and the protein expression of TGIF2, SOX2, CD133, CD44 and EGFR/MAPK signaling (D) in Scramble, TGIF2-OE, siSOX2 and TGIF2-OE/siSOX2 groups in PANC-1 cells. **E and F** The sphere number (E) and the protein expression of TGIF2, SOX2, CD133, CD44 and EGFR/MAPK signaling (F) in Scramble, siTGIF2, SOX2-OE and siTGIF2/SOX2-OE groups in BxPC-3 cells. (**G-H**) the spheroids number of PC in wildtype (treated with EGF to activate EGFR/ERK signaling) and Kras-mutant (constant activation of EGFR/ERK signaling) mice. (**I**) The overexpression of TGIF2, SOX2, CD133 and p-ERK were in pancreatic intraepithelial neoplasia (PanIN) of KC mice. **J.** Cell growth in Scramble, TGIF2-OE, siSOX2, TGIF2-OE/siSOX2 and TGIF2-OE/Erlotinib groups of PANC-1 cells and in Scramble, siTGIF2, SOX2-OE and siTGIF2/SOX2-OE of BxPC-3 cells by MTT.** K.** Under various concentration of Gemcitabine treatment, Cell proliferation rate in Scramble, TGIF2-OE, siSOX2, TGIF2-OE/siSOX2 and TGIF2-OE/Erlotinib groups of PANC-1 cells and in Scramble, siTGIF2, SOX2-OE and siTGIF2/SOX2-OE of BxPC-3 cells by MTT. Bars indicate ± S.E.*, P <0.05; **, P <0.01 compared with the control.

SOX2 plays a significant role in regulating tumor initiation and stem cell function. Thus, we investigated the role of the TGIF2/SOX2 axis in the self-renewal of CSCs using a sphere formation assay (Figure 4A-B, Figure S5D-E). The protein expression of TGIF2, SOX2, CD133, CD44, p-EGFR, and p-ERK was upregulated during the transition from monolayer cells to spheres, indicating a vital role for the TGIF2/SOX2 axis and EGFR/MAPK signaling in the self-renewal capacity of CSCs. Although SOX2 silencing partially decreased the number of spheres compared to the scramble group, TGIF-OE with SOX2 silencing rescued the decrease in the number of spheres caused by SOX2 silencing (Figure 4C). WB showed that TGIF2-OE promoted the expression of SOX2, CD133, EGFR, p-EGFR, and p-ERK, whereas SOX2 silencing partially downregulated the expression of these proteins. When TGIF2 overexpression and SOX2 silencing were performed concurrently, TGIF2-OE rescued the downregulated expression of SOX2, CD133, EGFR, p-EGFR, and p-ERK (Figure 4D and S5F). Conversely, TGIF2 silencing inhibited the self-renewal capacity of CSCs in BxPC-3 cells, which was significantly reversed by SOX2 overexpression (Figure 4E-F and S5G).

The initiation event of PC is caused by pancreatic intraepithelial neoplasia (PanIN). We found that TGIF2 and SOX2 silencing suppressed the number of spheroid pancreatic acinar cells in wild-type (treated with EGF to activate EGFR/ERK signaling) and KRAS-mutant (constant activation of EGFR/ERK signaling) mice. This trend was stronger in the TGIF2/SOX2 double-silenced group (Figure 4G-H). TGIF2, SOX2, CD133, and p-ERK were highly expressed in PanIN KC mice (6 months) (Figure 4I). All results indicated that the TGIF2/SOX2 axis not only promotes the self-renewal capacity of CSCs in PC by activating EGFR/MAPK signaling, but also participates in the early transformation events that occur in precancerous lesions.

With Gemcitabine treatment, the proliferation rate of TGIF2-OE PANC-1 cells was significantly higher than that in the scramble group. And SOX2 silencing partially inhibited cell proliferation and blunted the proliferation in the TGIF-OE group when SOX2 silencing was done concurrently (Figure 4J-K). Erlotinib, an inhibitor of EGF receptor (EGFR), also significantly inhibited TGIF2-OE induced Gemcitabine resistance, indicating a vital role of EGFR/MAPK signaling in TGIF2/SOX2 axis-mediated drug resistance. Conversely, SOX2-OE reversed TGIF2 inhibited Gemcitabine sensitivity in BxPC-3 cells (Figure 4J-K). Taken together, the coordination of TGIF2/SOX2 promotes CSCs and drug resistance in PC.

### TGIF2 with Smad2 activating SOX2 via Slug and EGFR expression

**Figure 5:**
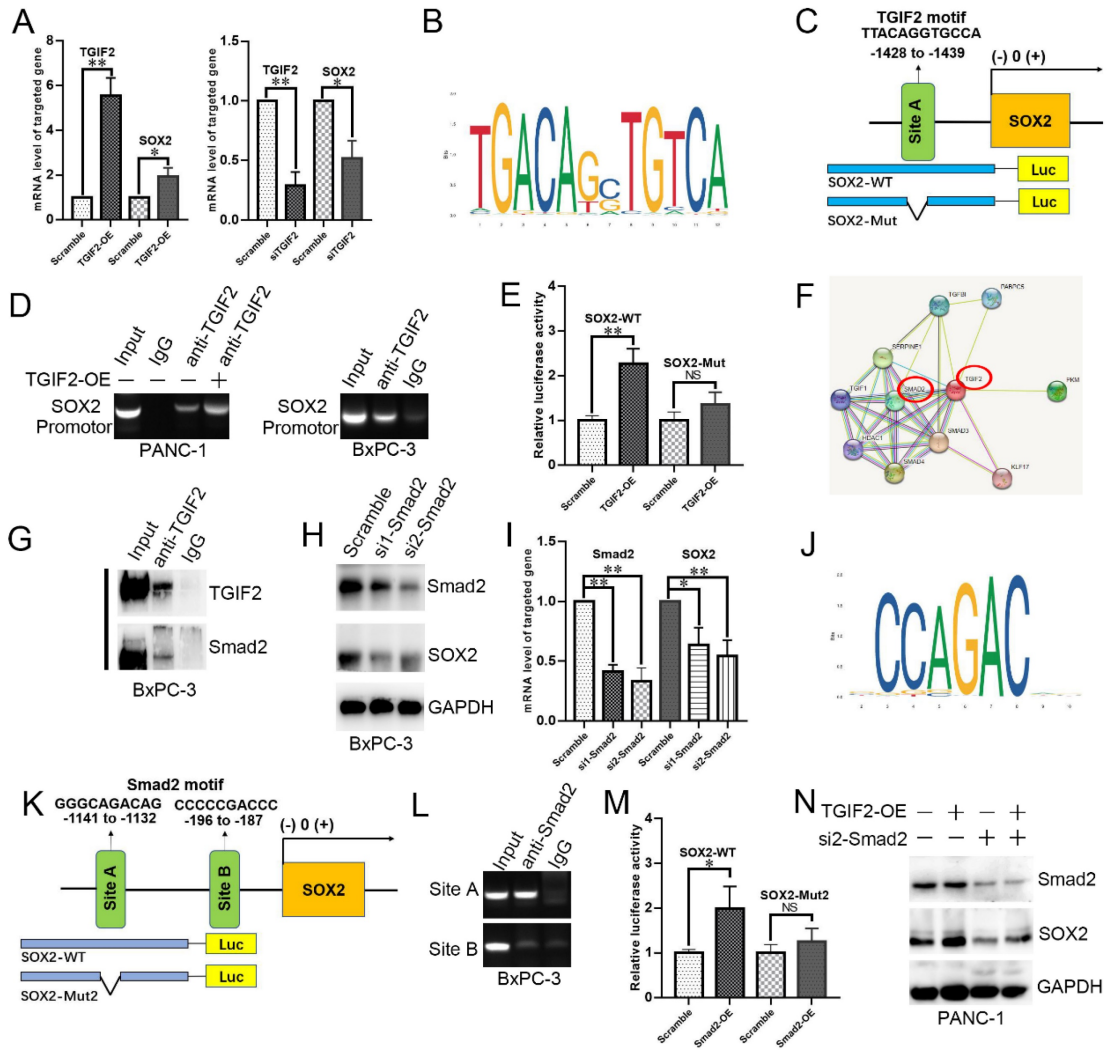
TGIF2 is a transactivation factor of SOX2 and interacts with Smad2 to co-regulate SOX2. **A.** The mRNA level of TGIF2 and SOX2 in TGIF2 overexpressing PANC-1 and TGIF2 silencing BxPC-3 cells, respectively. **B.** The binding site of TGIF2 in its zinc fingers domain was obtained from the JASPAR database. **C.** The predicted potential binding site of TGIF2 to SOX2 promoter, as well as wild-type/mutant SOX2 promoter plasmids (SOX2-WT or SOX2-Mut) designed accordingly.** D.** Chip assays in PANC-1 and BxPC-3 cells.** E.** Luciferase assay in 293 T cells co-transfected with SOX2-WT or SOX2-Mut promoter plasmid and TGIF2 overexpression plasmid. **F.** The protein interaction network of TGIF2 via String database. **G.** Co-IP was performed in PANC-1 and BxPC-3 cells.** H and I.** The protein and mRNA level of Smad2 and SOX2 in Smad2 silencing BxPC-3 cells. **J.** The binding site of Smad2 in its zinc fingers domain was obtained from the JASPAR database.** K.** The predicted potential binding site of Smad2 to SOX2 promoter, as well as wild-type/mutant SOX2 promoter plasmids (SOX2-WT or SOX2-Mut2) designed accordingly. **L.** Chip assays in BxPC-3 cells.** M.** Luciferase assay in 293 T cells co-transfected with SOX2-WT or SOX2-Mut2 promoter plasmid and Smad2 overexpression plasmid (or empty vector scramble). **N.** The protein level of Smad2 and SOX2 in Scramble, TGIF2-OE, si2-Smad2 and TGIF2-OE/si2-Smad2 groups in PANC-1 cells. Bars indicate ± S.E.*, P <0.05; **, P <0.01 in contrast with the control.

We first showed that TGIF2-OE or silence also upregulated or downregulated the mRNA level of SOX2, respectively (Figure 5A). The binding site of TGIF2 on SOX2 was obtained from the JASPAR database (Figure 5B). We designed a specific primer to amplify the SOX2 promoter and the corresponding wild-type SOX2 (SOX2-WT) and mutant SOX2 (SOX2-Mut) plasmids (Figure 5C). Upon immunoprecipitation with an anti-TGIF2 antibody, the DNA fragment containing site A was amplified to a significantly higher level. The amplification was more significant in TGIF2 overexpression group (Figure 5D). Furthermore, the luciferase activity of the SOX2-WT promoter was significantly stimulated by the co-transfection of TGIF2-OE in HEK-293 cells (Figure 5E). However, when the binding site of TGIF2 on SOX2 was mutated, the activating effect was markedly attenuated (Figure 5E). Thus, TGIF2 transactivates SOX2 in PC cells.

The protein interaction network of TGIF2 showed strong interaction TGIF2 with Smad2 and Smad3 (Figure 5F). Co-IP further verified that TGIF2 and Smad2 co-immunoprecipitated in anti-TGIF2 cells (Figure 5G and S5H). Smad2 silencing attenuated SOX2 expression, indicating that Smad2 may be a potential TF for SOX2 (Figure 5H-I, Figure S5I). We predicted Smad2, but not Smad3, as the potential TF of SOX2 using the hTFtarget and Jaspar databases (Figure 5J) and designed the corresponding binding sites in SOX2-WT and SOX2-Mut (Figure 5K). After pull-down with anti-Smad2, the DNA fragment containing site A, but not site B, was amplified at a significantly higher level from the chromatin of BxPC-3 cells (Figure 5L). Additionally, luciferase activity of the SOX2-WT promoter was significantly stimulated by co-transfection with Smad2-OE in HEK-293 cells (Figure 5M). This activating effect was markedly attenuated (Figure 5M) when the binding site of Smad2 to SOX2 was mutated. Moreover, the upregulation of SOX2 induced by TGIF2-OE was significantly reversed by Smad2 silencing (Figure 5N and S5J), further indicating that Smad2 influences TGIF2 induced SOX2 expression.

**Figure 6:**
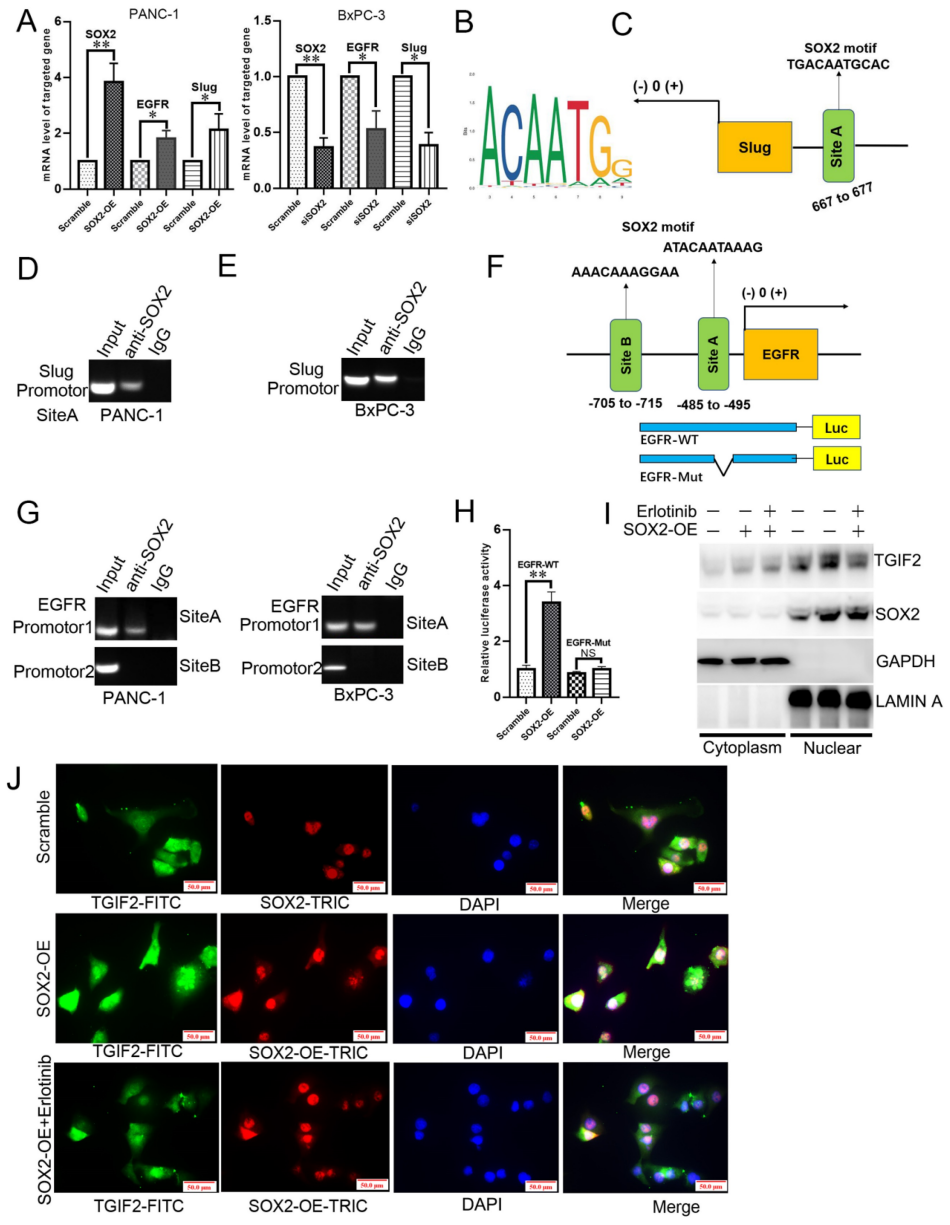
SOX2 is a transactivation factor of EGFR and Slug. **A.** The mRNA level of SOX2, EGFR and Slug in SOX2 overexpressing PANC-1 and SOX2 silencing BxPC-3 cells, respectively. **B.** The DNA binding site of SOX2 was obtained from the JASPAR database. **C.** The predicted potential binding site of SOX2 to Slug promoter.** D and E.** Chip assays in PANC-1 (D) and BxPC-3 (E) cells. **F.** The predicted potential binding site of SOX2 to EGFR promoter, as well as wild-type/mutant EGFR promoter plasmids designed accordingly. **G.** Chip assays in PANC-1 and BxPC-3 cells. **H.** Luciferase assay in 293 T cells co-transfected with EGFR-WT (or EGFR-Mut) promoter plasmid and SOX2 overexpression plasmid (or empty vector corresponding to scramble group). **I.**The roles of SOX2-OE and Erlotinib treatment to TGIF2 nuclear translocation. **J.** IF staining of TGIF2 and SOX2 in Scramble, SOX2-OE and SOX2-OE plus Erlotinib groups of PANC-1. Bars indicate ± S.E.*, P <0.05; **, P <0.01 in contrast with the control.

SOX2-OE or silence upregulated or downregulated the mRNA levels of EGFR and Slug, respectively, in PANC-1 and BxPC-3 cells (Figure 6A). The slug-binding site is shown in Figure 6B, and a specific primer for the slug promoter was obtained (Figure 6C). Upon anti-SOX2 treatment, the DNA fragment containing site A was amplified at a significantly higher level from the chromatin of PANC-1 and BxPC-3 cells (Figure 6D-E). The predicted binding sites in the EGFR promoter (sites A and B) are shown in Figure 6F. The amplification of site A was significantly higher than that of site B in the chromatin of cells (Figure 6G), revealing that TGIF2 is a transcriptional activator of SOX2, which in turn promotes EMT and CSCs by transactivating Slug and EGFR expression. Moreover, the luciferase activity was augmented in the EGFR-WT promoter plasmid rather than in EGFR-Mut, which further verified the specific binding site of SOX2 to the EGFR promoter (Figure 6H).

Notably, WB revealed that SOX2 overexpression promoted TGIF2 nuclear translocation in PANC-1 cells, which was reversed by erlotinib treatment (Figure 6I). IF further showed that TGIF2 and SOX2 colocalized in the cytoplasm and nucleus of PANC-1 cells. SOX2 overexpression promoted TGIF2 nuclear translocation, which was reversed by erlotinib treatment (Figure 6J). Thus, transactivation of SOX2 by TGIF2 promotes EGFR/MAPK signaling, which in turn promotes TGIF2 nuclear translocation, forming a positive feedback loop *in vitro*. Moreover, the binding sites of TGIF2/Smad2 with SOX2 were detected in ESC and DE at three time points: ESC, DE-24h, and DE-48h. The openness and promoters of the three genes were activated and increased with time, indicating the overlap of ATAC-seq peaks among genes (Figure S4J).

### Coordination of TGIF2/SOX2 promotes tumor and liver metastasis

**Figure 7:**
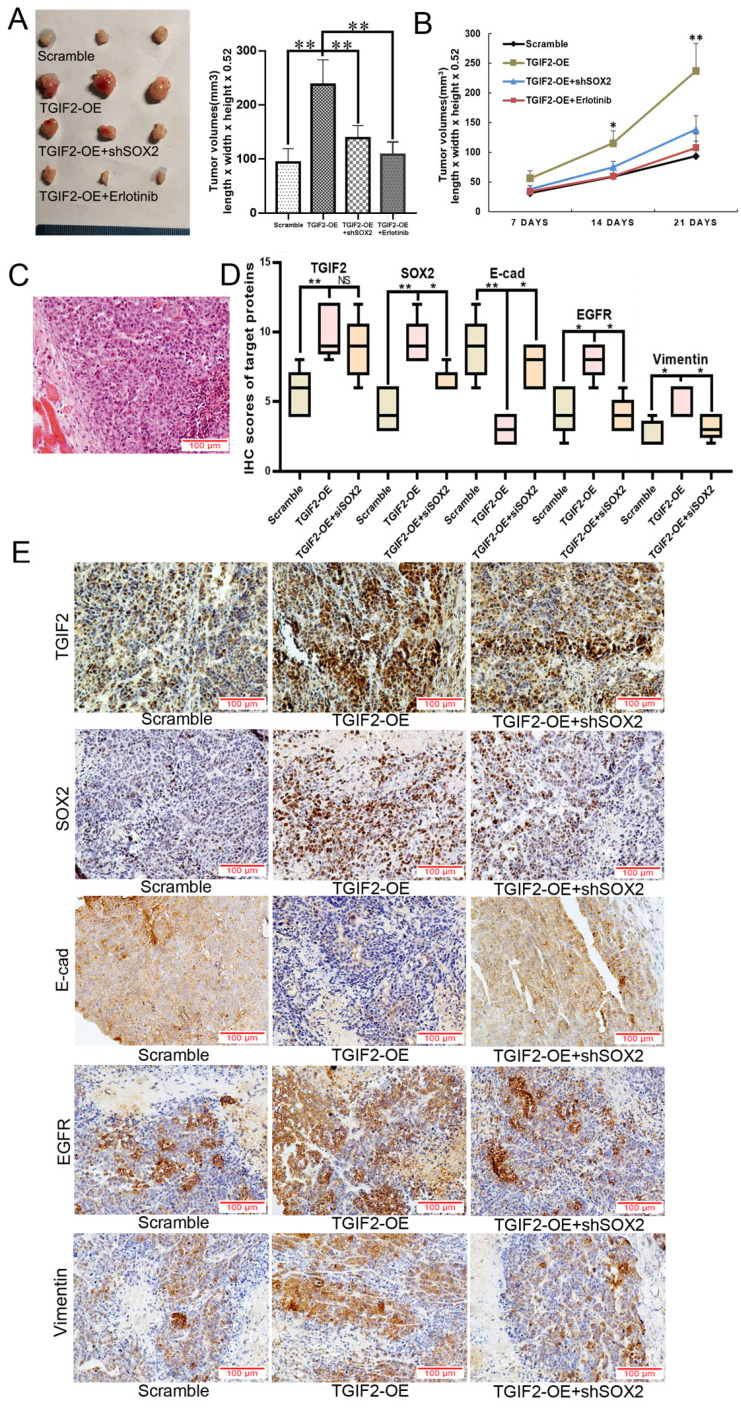
Coordination of TGIF2/SOX2 promotes subcutaneous tumor size *in vivo*. **A.** Tumor volumes in Scramble, TGIF2-OE, TGIF2-OE/shSOX2, and TGIF2-OE plus Erlotinib groups implanted with PANC-1 cells.** B.** Tumor growth curve in above group. **C.** HE staining of harvested tumor.** D.** The statistical data of IHC assays. **E.** The different expression of TGIF2, SOX2, E-cad, EGFR and Vimentin in Scramble, TGIF2-OE, and TGIF2-OE/shSOX2 groups by IHC. Bars indicate ± S.E. *, P<0.05; **, P<0.01 compared with control.

As shown in Figure 7A-B, the subcutaneous tumors in the TGIF2-OE group were much larger than those in the scramble group in a time-dependent manner. However, SOX2 silencing or erlotinib treatment significantly reversed TGIF2 overexpression-promoted increase in tumor size (TGIF2-OE vs. TGIF2-OE/shSOX2; TGIF2-OE vs TGIF2-OE/Erlotinib) (Figure 7A-B). Hematoxylin and eosin (HE) staining confirmed the tumor pathology (Figure 7C). IHC verified that the expression of TGIF2, SOX2, EGFR, and Vimentin was increased and that of E-cadherin was decreased in the TGIF2-OE group compared with the scramble group and reversed in the TGIF2-OE combined with shSOX2 group (Figure 7D-E).

**Figure 8:**
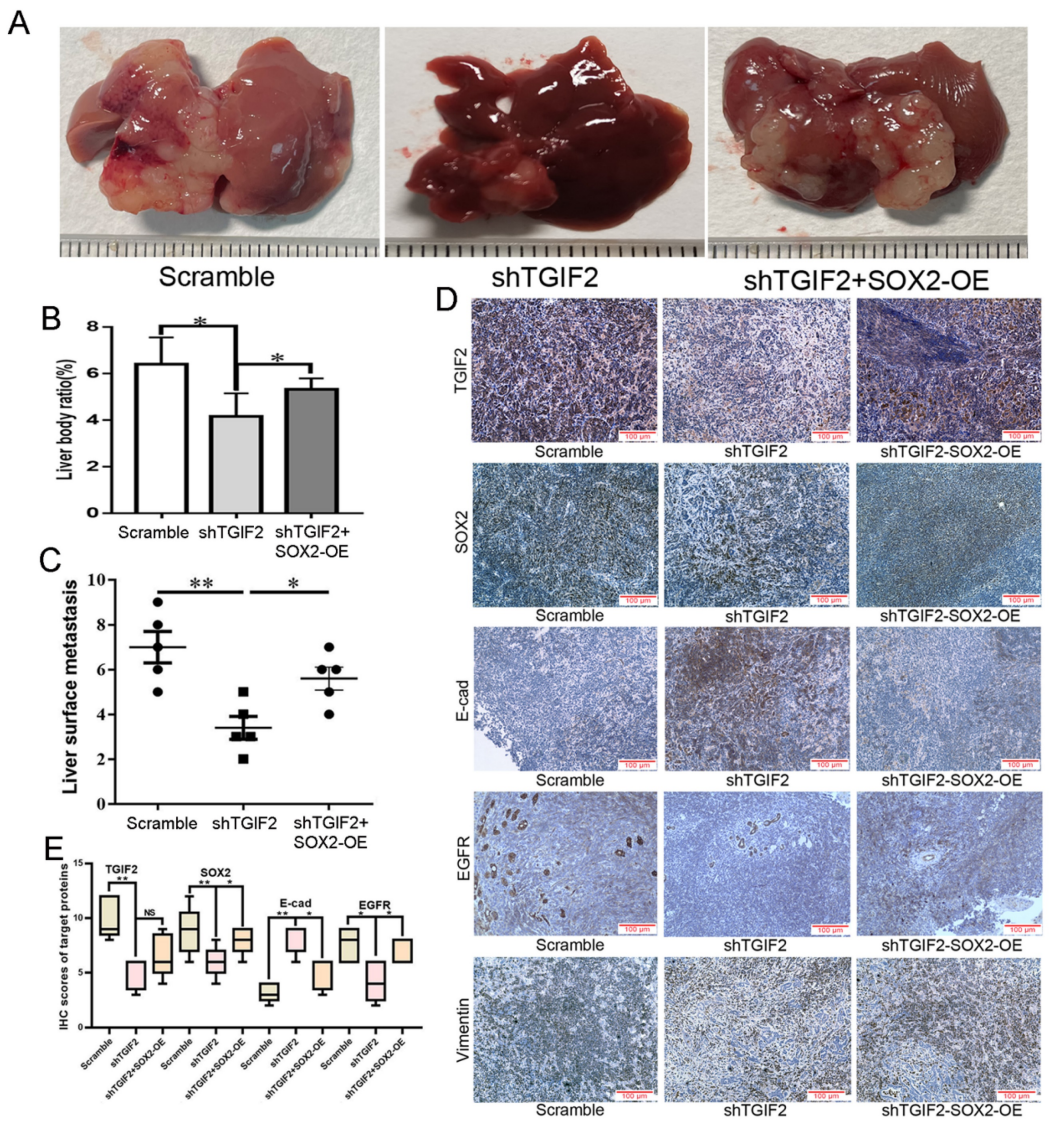
Coordination of TGIF2/SOX2 promotes liver metastasis *in vivo*.** A**. Liver metastasis in Scramble, shTGIF2 and shTGIF2/SOX2-OE groups. **B-C.** The Liver body ratio and the number of liver metastases in above groups. **D.** The different expression of TGIF2, SOX2, E-cad, EGFR and Vimentin in Scramble, shTGIF2 and shTGIF2/SOX2-OE groups by IHC. **E.** The statistical data of IHC assays. Bars indicate ± S.E. *, P<0.05; **, P<0.01 compared with control.

**Figure 9:**
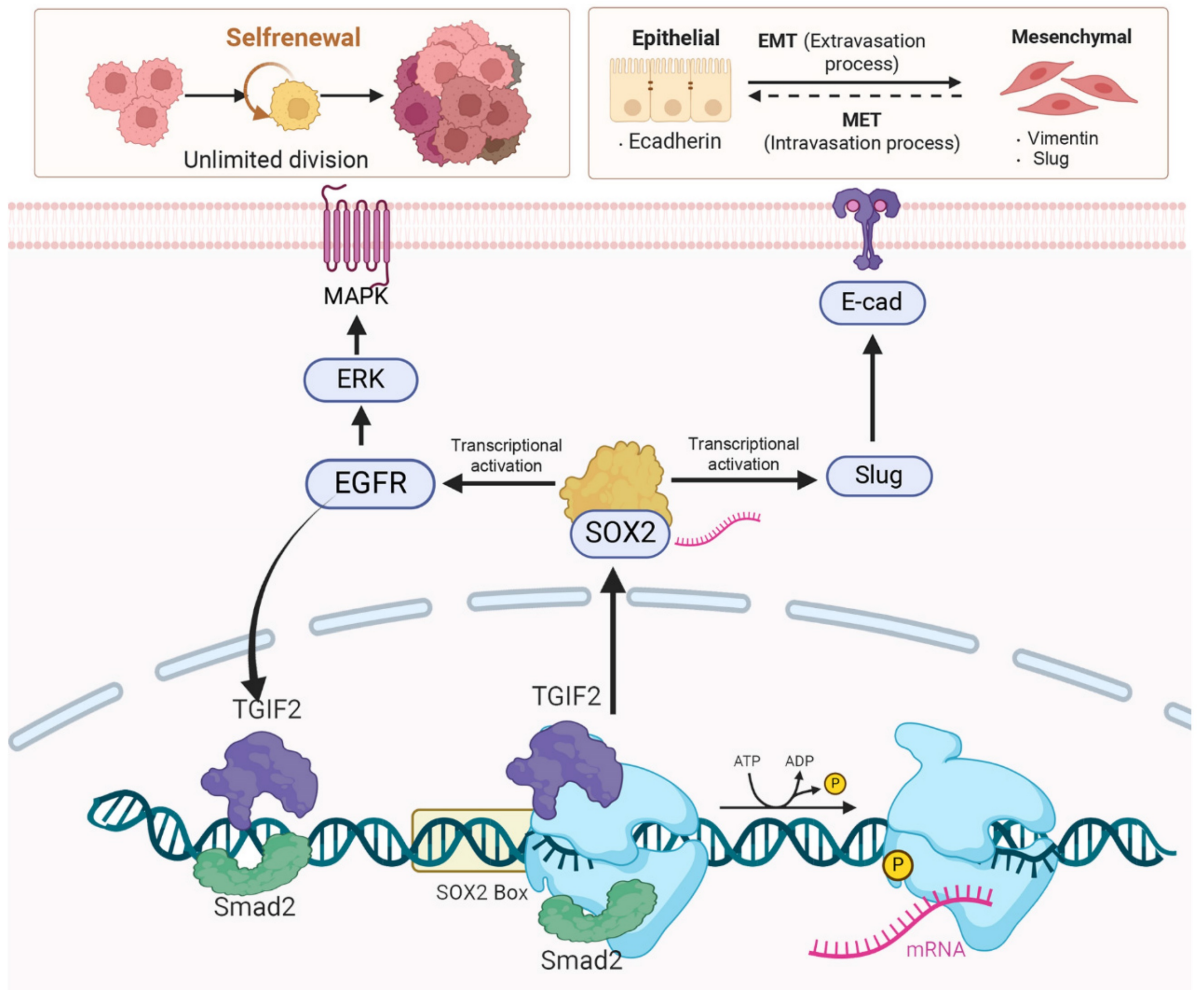
Smad2 cooperating with TGIF2 contributes to CSC and EMT via co-targeting SOX2. TGIF2 activates SOX2 promoter via interacting with Smad2, which stimulates EMT and EGFR/MAPK signaling by transactivating Slug and EGFR, and promoting EMT and CSCs function. Moreover, the stimulation of EGFR/MAPK signaling by SOX2 promotes TGIF2 nuclear translocation, forming a positive feedback loop.

The number of liver metastases and the corresponding liver body ratio in TGIF2 silencing group (shTGIF2 group) were much lower than those in the scrambled control group (Figure 8A-B). However, SOX2 overexpression (SOX2-OE) significantly reversed TGIF2 silencing, which inhibited the decrease in liver metastasis and the corresponding liver body ratio (shTGIF2 group vs. shTGIF2/SOX2-OE group) (Figure 8A-C). Liver body IHC further verified that TGIF2, SOX2, EGFR, and Vimentin were decreased, but E-cadherin was increased in the shTGIF2 group compared with the scramble group, which was significantly reversed in the shTGIF2/SOX2-OE group (Figure 8D-E). Taken together, the coordination of TGIF2/SOX2 promotes subcutaneous tumor size and liver metastasis by activating EMT and EGFR/MAPK signaling (Figure 9).

## Discussion

However, the transcriptional regulatory network of CSC and EMT in PC progression is poorly understood. In the present study, TGIF2 and Smad2 were identified as novel SOX2 transcription factors. Transactivation of SOX2 through cooperation between TGIF2 and Smad2 transcriptionally activates Snail2 and EGFR, which in turn promotes EMT and CSC. Meanwhile, SOX2 activates EGFR-ERK/MAPK signaling, promoting TGIF2 nuclear translocation and forming a positive feedback loop, which has not been reported, to our knowledge.

In this study, we identified TGIF2 as a key player in the CSC signature using scRNA analysis and machine learning. TGIF2 overexpression indicates advanced clinical stage and poor prognosis. We then explored eight targets of TGIF2, and SOX2 showed strong correlations. The clinical relevance of SOX2 in PC was consistent with that of TGIF2, which also prevailed of TGIF2 in lung cancer and cervical cancer 47, 48 and SOX2 in cervical cancer 49, colorectal cancer 50, and urethral carcinoma 51. TGIF2 was positively correlated with SOX2 and EGFR, but negatively associated with E-cadherin expression, and the cooperation of TGIF2 and SOX2 contributed to a worse prognosis for patients with PC. These findings indicate a close interaction between the TGIF2/SOX2 axis in CSC and EMT of PC, which prompted us to further explore the mechanism.

Moreover, TGIF2-OE induced an EMT-like phenotype following an increase in SOX2, Vimentin and Slug expression and a decrease in E-cadherin expression, which was reversed by SOX2 silencing. In lung cancer, TGIF2 phosphorylation is a therapeutic target that drives EMT and metastasis 48. However, TGIF2 was reported to repress EMT of oral squamous cell carcinoma 25. The dual role of TGIF2 in EMT may be due to differences in cancer types and microenvironments, which helps us understand the general or cancer-type-specific EMT response. Specifically, genomic alterations in EMT have been systematically studied in gastric cancer, which is related to the fact that the stomach and pancreas are both endoderm-derived tissues that share some TFs. We have previously investigated the molecular mechanisms mediating the initiation and development of EMT in PC 10-13, 52. Various key regulators (CALR, MSI2-Numb, ZNF263/ZNF31, and GINS2) play significant roles in promoting EMT following genomic alterations under different tumor microenvironment conditions. Most of these genes also participate in EMT in GC 53-56. Similarly, common EMT gene signatures found in GC also exist in PC, including ZEB1, RUNX and AP-1^36^-^38^. For example, Zeb1 induces PC plasticity as an EMT activator 13, RUNX acts as a master transcription factor in PC 57, and AP-1 regulates the activation of Akt signaling during PC tumorigenesis 58.

Dormant cells stimulate EMT phenotypes to promote the metastasis in CSC-like phenotypes 51, which is key in cancer drug resistance acquisition and malignant plasticity 59. Our findings showed that TGIF2-OE promoted the self-renewal capacity of CSCs and drug resistance following the upregulation of SOX2, CD133, CD44, and EGFR/ERK signaling, which was also reversed by SOX2 silencing. PanIN is one of the earliest events involved in exocrine PC development 48, 60. Oncogenic KrasG12D inhibits healing progression by blocking redifferentiation and promoting PanIN formation 61. Here, we found that the TGIF2/SOX2 axis induces PanIN by increasing the number of acinar-derived spheroids in KRAS-mutant mice. Thus, the TGIF2/SOX2 axis not only promotes the self-renewal capacity of CSCs but also participates in the early events of PC involving the initiation and progression of PanIN.

Mechanistically, TGIF2 immunoprecipitated with Smad2 and induced the transcription of SOX2. Smad2, but not Smad3, acts as a parallel transcriptional activator of SOX2 by binding to its promoter. This difference between Smad2 and Smad3 in DNA binding is likely due to a sequence insert in the N domain of Smad2, immediately before the DNA binding β hairpin, which might be a block of DNA recognition 62. TGIF interacted with Smad2/3 in a TGF-β-inducible manner, resulting in the recruitment of TGIF to Smad-responsive DNA elements 63. Though TGIF2 interacts with TGF-β-activated Smads and represses TGF beta-responsive transcription, it appears to be a context-dependent transcriptional activator or repressor consistent with other TALE homeoproteins 23. For example, TGIF2 promotes lung cancer stemness by transactivating OCT4 48. It can also bind to the CDH1 promoter and activate CDH1 expression in epithelial cells of colon cancer cells 64. The mechanism of transcriptional activation by Smad is most likely defined by the combined requirement of interactions with co-transcription activators and promoter DNA sequences. In the current study, Smad2 cooperated with TGIF2 to promote the transcriptional activity of SOX2. Smad2 is key for maintaining the pluripotent stem cell state 65. For example, Smad2/3 is previously known to directly binds and regulates the expression of NANOG to sustain human ESC self-renewal 66. ChIPseq in human ESCs also revealed the binding of SMAD2/3 to OCT4, TERT, MYC, and DPPA4 67. Here, Smad2 was first identified as a transcriptional activator of SOX2 via its interaction with TGIF2, which, to the best of our knowledge, has not yet been clarified.

Transactivation of SOX2 has also been identified as a novel TF activator of Slug and EGFR via binding to their promoters. The transcription factor Slug represses E-cadherin expression and induces EMT in several cancers 68, while EGFR/MAPK signaling promotes CSC function in colorectal, breast, and squamous cell carcinoma 69-71. Thus, the TGIF2/SOX2 axis promotes EMT and CSCs properties via activation of Slug and EGFR signaling. Interestingly, the transactivation of EGFR/MAPK signaling by SOX2 promotes TGIF2 nuclear translocation, forming a positive feedback loop. EGFR-RAS-ERK signaling phosphorylates TGIF2 and increases its stability 48. TGIF2 also stimulates EGFR/MAPK signaling by activating SOX2, which, in turn, increases TGIF2 stability by promoting its nuclear translocation.

Taken together, TGIF2 and smad2 targeting SOX2 promoted EMT and CSCs properties via the activation of Slug and EGFR signaling, respectively. The transactivation of EGFR/MAPK signaling by SOX2 promotes TGIF2 nuclear translocation, forming a positive feedback loop (Figure 9). The TGIF2/SOX2 transcriptional axis contributes to EMT, cancer stem cell properties, and chemoresistance in PC and is a promising target for PC therapy.

## Supplementary Material

Supplementary figures and tables.

## Figures and Tables

**Table 1:** The relationship among TGIF2, SOX2, EGFR and E-cad expression in 88 cases of clinical PC samples

Parameters		TGIF2		*r*	*P*
		Low	High		
SOX2	Low	33	20	0.330	0.002
	High	10	25		
EGFR	Negative	36	27	0.263	0.014
	Positive	7	18		
E-cad	Abnormal	21	32	-0.227	0.033
	Normal	22	13		

**Table 2:** The clinicopathological significance of TGIF2 and SOX2 expression in 88 cases of clinical PC samples

Parameters	No. of patients	TGIF2		*P*	SOX2		*P*
		Low	High		Low	High	
Cases	88	43	45		53	35	
Age (years)							
≤65	51	24	27	0.691	31	20	0.900
>65	37	19	18		22	15	
Gender							
Male	57	25	32	0.203	32	25	0.288
Female	31	18	13		21	10	
Tumor size (cm)							
<3	35	21	14	0.127	26	9	0.029
≥3	53	22	31		27	26	
Tumor location							
Head	62	33	29	0.206	35	27	0.264
Body-tail	26	10	16		18	8	
Differentiation							
Well	35	20	15	0.207	23	12	0.393
Moderate to Poor	53	23	30		30	23	
T stage ^a^							
T1+T2	43	27	16	0.011	31	12	0.026
T3	45	16	29		22	23	
LN metastasis ^b^							
N0 (negative)	60	34	26	0.032	38	22	0.383
N1 (positive)	28	9	19		15	13	
UICC stage ^a^							
I+IIA stage	62	35	27	0.028	40	22	0.204
IIB+III stage	26	8	18		13	13	
Vascular permeation							
Absent	50	28	22	0.124	31	19	0.697
Present	38	15	23		22	16	

a. According to 8th TNM stage of AJCC. b. Lymph node

**Table 3:** Univariate and Multivariate analysis in survival time

Parameters	Median survival (months)	Univariate analysis *P* (log rank)	Multivariate analysis hazard ratio (95% CI)	*P*
Differentiation				
(Well/Moderate to Poor)	31.8 vs 21.8	0.065	-	-
T stage				
(T1+T2/T3)	30.3 vs 21.1	0.072	-	-
Lymph nodes metastasis (N0/N1)	31.3 vs 19.4	0.002	1.39(0.61-3.11)	0.430
Vascular permeation (absent/present)	31.8 vs 18.8	0.004	1.62 (0.73-3.60)	0.233
UICC stage	30.3 vs 17.8	0.002	2.39(1.24-4.56)	0.008
(I+IIA/IIB+III)				
TGIF2				
(Low/High)	34.1 vs 21.7	0.002	2.16(1.10-4.24)	0.024
SOX2				
(Low/High)	27.4 vs 21.7	0.100	-	-
E-cad				
(Normal/Abnormal)	27.5 vs 23.1	0.240	-	-
EGFR				
(Negative/Positive)	27.4 vs 22.1	0.123	-	-

## Abbreviations

EMT: Epithelial-mesenchymal transition
CSC: cancer stem cell
scRNA: Single-cell RNA
WGCNA: weighted gene co-expression network
t-SNE: t-Distributed Stochastic Neighbor Embedding
TCGA: The Cancer Genome Atlas
GEO: Gene Expression Omnibus
Gastric cancer: GC
Pancreatic cancer: PC
TGIF2: TGF-β-induced factor homeobox 2
SOX2: sex determining region Y box 2
Smad2: SMAD family member 2
ERK: Extracellular regulated protein kinases
EGFR: epidermal growth factor receptor
MAPK: mitogen-activated protein kinase
E-cad: E-cadherin
N-cad: N-cadherin
