# Supplementary material for: Smad2 Cooperating with TGIF2 Contributes to EMT and Cancer Stem Cells Properties in Pancreatic Cancer via Co-Targeting SOX2
Source: Int J Biol Sci. 2025 Jan 1;21(2):524–43. doi: 10.7150/ijbs.102381 (PMC11705628; doi:10.7150/ijbs.102381)
Supplement: Supplementary file 1 — Supplementary figures and tables. [file ijbsv21p0524s1.pdf]

Supplementary Figure legends:

Figure S1. Landscape of CSC signature in PC patients. **A.** The screening criteria of scRNA in PC samples. **B.** The distribution of six CSC genes in stem cell sub-clusters. **C.** All the risk models were identified in PC via NFM algorithm. **D.** Two categories calculated the risk score of PC using ssGSEA algorithm. **E.** CSC genes are identified in stem genes and subtype genes. **F and G.** The mRNA level of TGIF2 and SOX2 in PC and normal pancreas according to GEPIA database (T: PC; N: pancreas). **H.** Heatmap plot of CSC genes in the expression level of subtypes.

Figure S2. The identification and validation of machine learning model. **A** The C-index of each model in four databases based on a machine learning model. **B and C.** The 1-, 3-, and 5-year survival of patient's scores both in E-MTAB and TCGA. **D and E**. The value of CSC model in univariate and multivariate analysis both in E-MTAB and TCGA. **F and G.** The nomogram was established to predict the prognosis, and the overall survival of 1-, 3-, and 5-year was validated via calibration plots and decision curve analysis (DCA) of a nomogram prediction.

Figure S3. The enriched pathway in CSC genes. **A-B.** The functional enriched analysis of CSC genes in E-MTAB cohort and TCGA cohort. **C.** The combination of functional pathways in E-MTAB cohort. **D-G.** The survival difference between the high and low-risk groups based on KRAS SIGNALING and EPITHELIAL MESENCHYMAL TRANSITION. **H.** The correlation plot of TGIF2 associated with EMT Key regulator Snail2 (Slug) and EGFR expression in TCGA database.

Figure S4. The binding of TGIF2 and SOX2. **A.** The target genes of TGIF2 through Stem genes and TFDB. **B and C.** The EMT signatures of gastric cancer and PC. **D-I.** The correlation between TGIF2 with target genes including MYC, SOX2, TP53, LMNB1, YY1, OTX2. **J.** The Chromatin openness among TGIF2, SOX2 and Smad2 in three represent time including ESC, DE-24h and DE-48h.

Figure S5. All the raw images of Western blot in our manuscript. **A.** The Western blot and statistical analysis of Figure 3A. **B-C.** The Western blot and statistical analysis of Figure 3C and Figure 3D respectively. **D-G.** The Western blot and statistical analysis of Figure 4A, Figure 4B, Figure 4D and Figure 4F, respectively. **H.** The Western blot and statistical analysis of Figure 5G. **I.** The Western blot and statistical analysis of Figure 5H. **J.** The Western blot and statistical analysis of Figure 5N.

Supplementary Tables legends:

Table S1. Primers used in this study

Table S2. siRNA sequences for TGIF2 and SOX2

Table S3. The clinicopathological significance of in 20 cases in PC

Table S4. Common targets of TGIF2 in TFDB 3.0 database.

Figure S1. Landscape of CSC signature in PC patients. **A.** The screening criteria of scRNA in PC samples. **B.** The distribution of six CSC genes in stem cell sub-clusters. **C.** All the risk models were identified in PC via NFM algorithm. **D.** Two categories calculated the risk score of PC using ssGSEA algorithm. **E.** CSC genes are identified in stem genes and subtype genes. **F and G.** The mRNA level of TGIF2 and SOX2 in PC and normal pancreas according to GEPIA database (T: PC; N: pancreas). **H.** Heatmap plot of CSC genes in the expression level of subtypes.

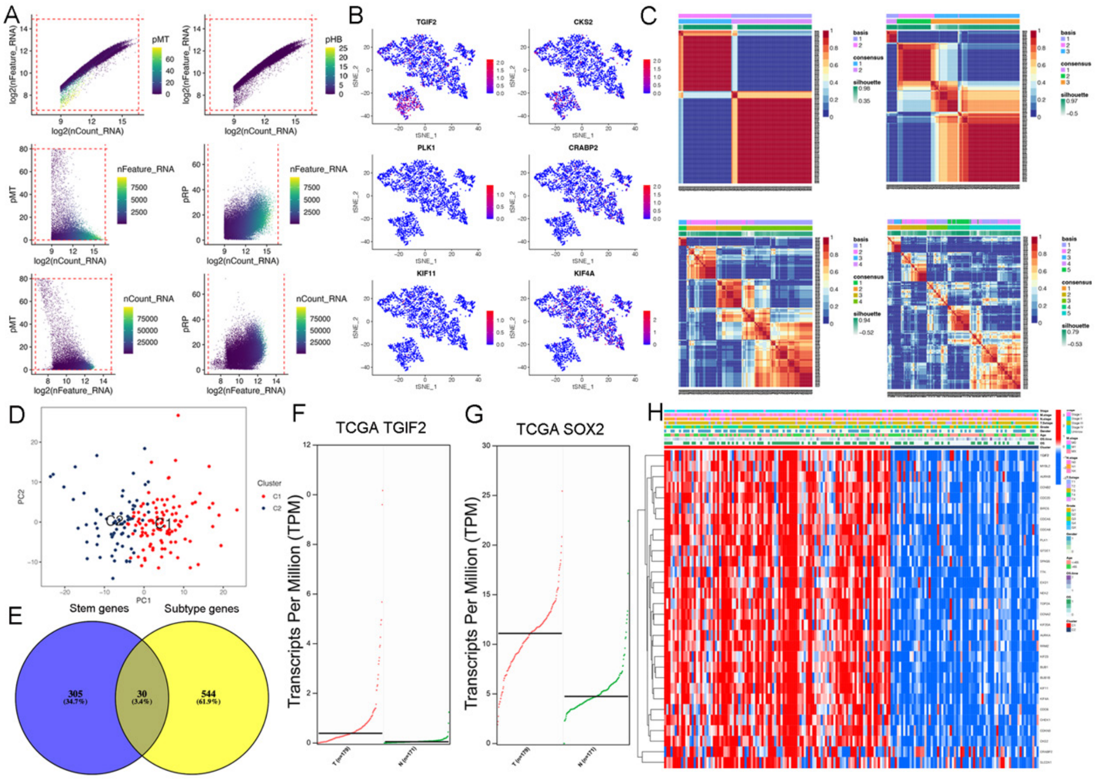

Figure S2. The identification and validation of machine learning model. **A** The C-index of each model in four databases based on a machine learning model. **B and C.** The 1-, 3-, and 5-year survival of patient's scores both in E-MTAB and TCGA. **D and E**. The value of CSC model in univariate and multivariate analysis both in E-MTAB and TCGA. **F and G.** The nomogram was established to predict the prognosis, and the overall survival of 1-, 3-, and 5-year was validated via calibration plots and decision curve analysis (DCA) of a nomogram prediction.

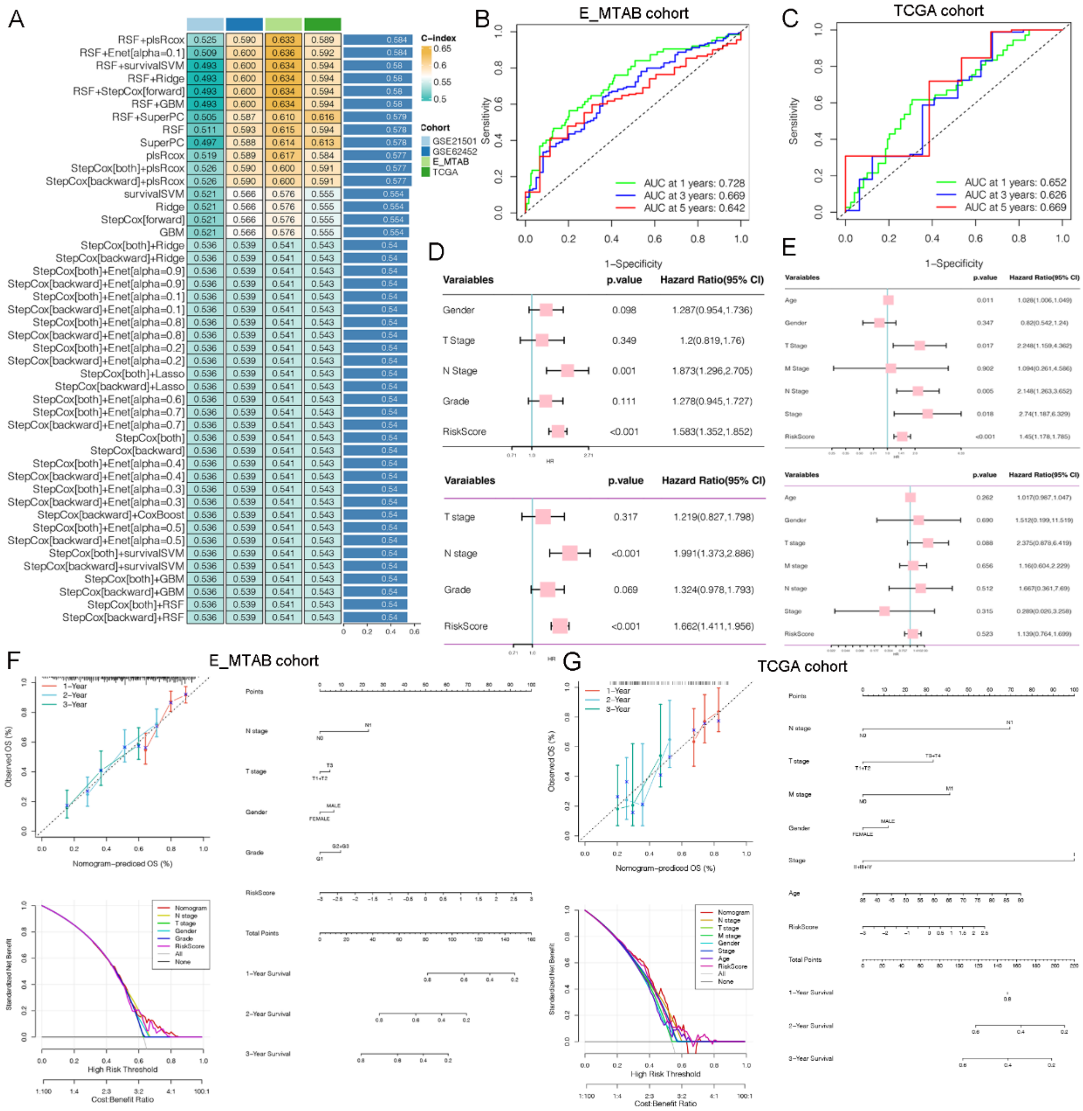

Figure S3. The enriched pathway in CSC genes. **A-B.** The functional enriched analysis of CSC genes in E-MTAB cohort and TCGA cohort. **C.** The combination of functional pathways in E-MTAB cohort. **D-G.** The survival difference between the high and low-risk groups based on KRAS SIGNALING and EPITHELIAL MESENCHYMAL TRANSITION. **H.** The correlation plot of TGIF2 associated with EMT Key regulator Snail2 (Slug) and EGFR expression in TCGA database.

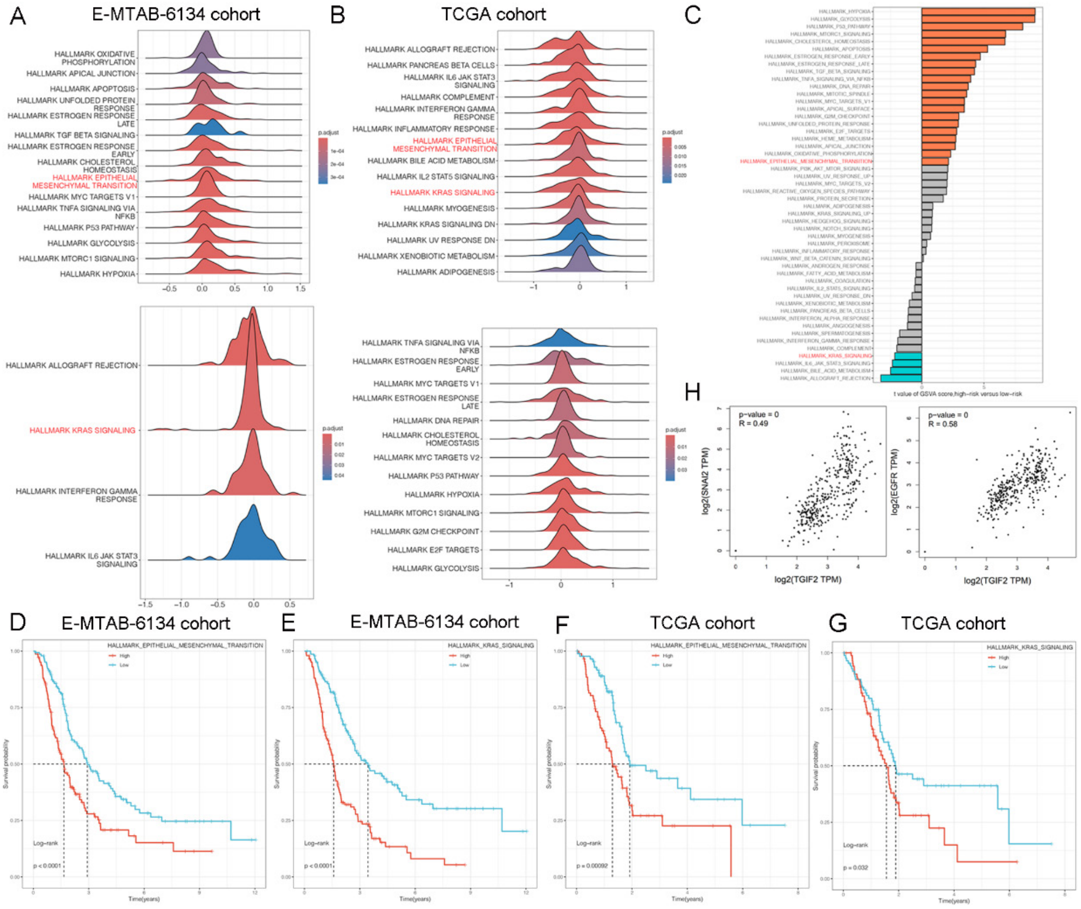

Figure S4. The binding of TGIF2 and SOX2. **A.** The target genes of TGIF2 through Stem genes and TFDB. **B and C.** The EMT signatures of gastric cancer and PC. **D-I.** The correlation between TGIF2 with target genes including MYC, SOX2, TP53, LMNB1, YY1, OTX2. **J.** The Chromatin openness among TGIF2, SOX2 and Smad2 in three represent time including ESC, DE-24h and DE-48h.

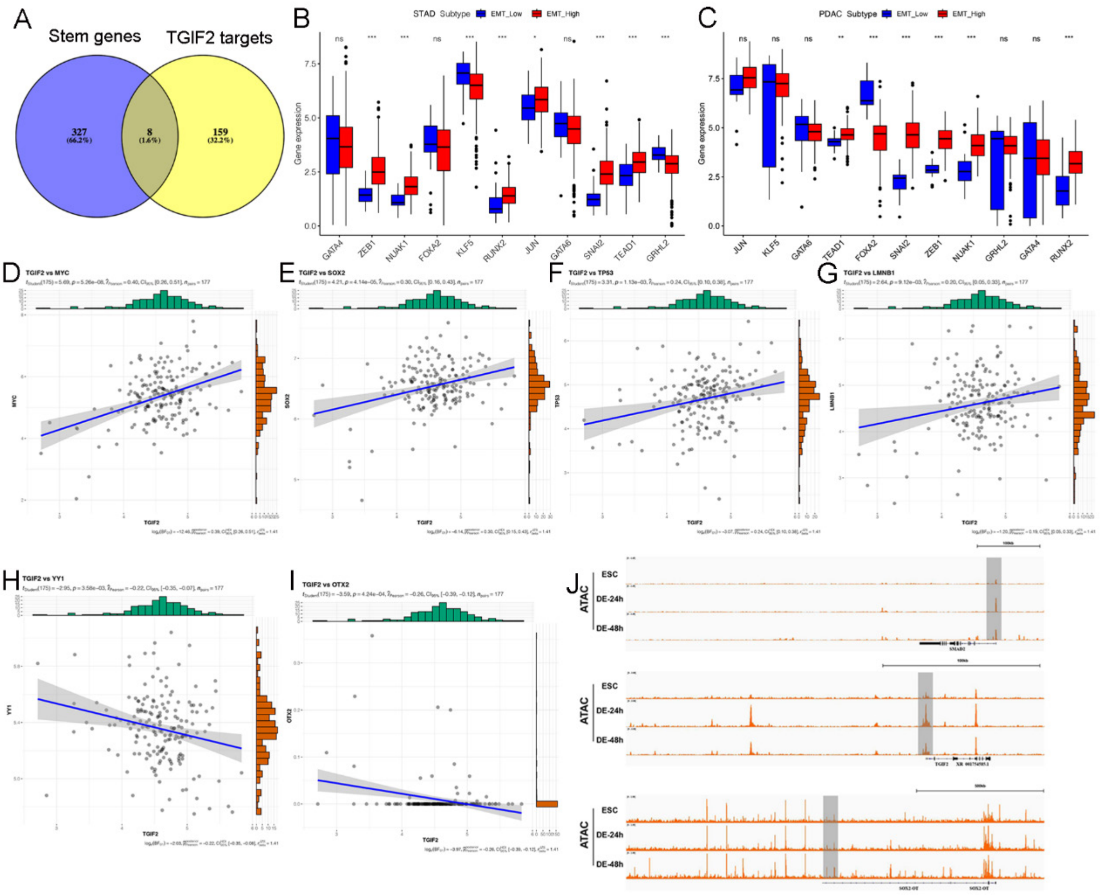

Figure S5. All the raw images of Western blot in our manuscript. **A.** The Western blot and statistical analysis of Figure 3A. **B-C.** The Western blot and statistical analysis of Figure 3C and Figure 3D respectively. **D-G.** The Western blot and statistical analysis of Figure 4A, Figure 4B, Figure 4D and Figure 4F, respectively. **H.** The Western blot and statistical analysis of Figure 5G. **I.** The Western blot and statistical analysis of Figure 5H. **J.** The Western blot and statistical analysis of Figure 5N.

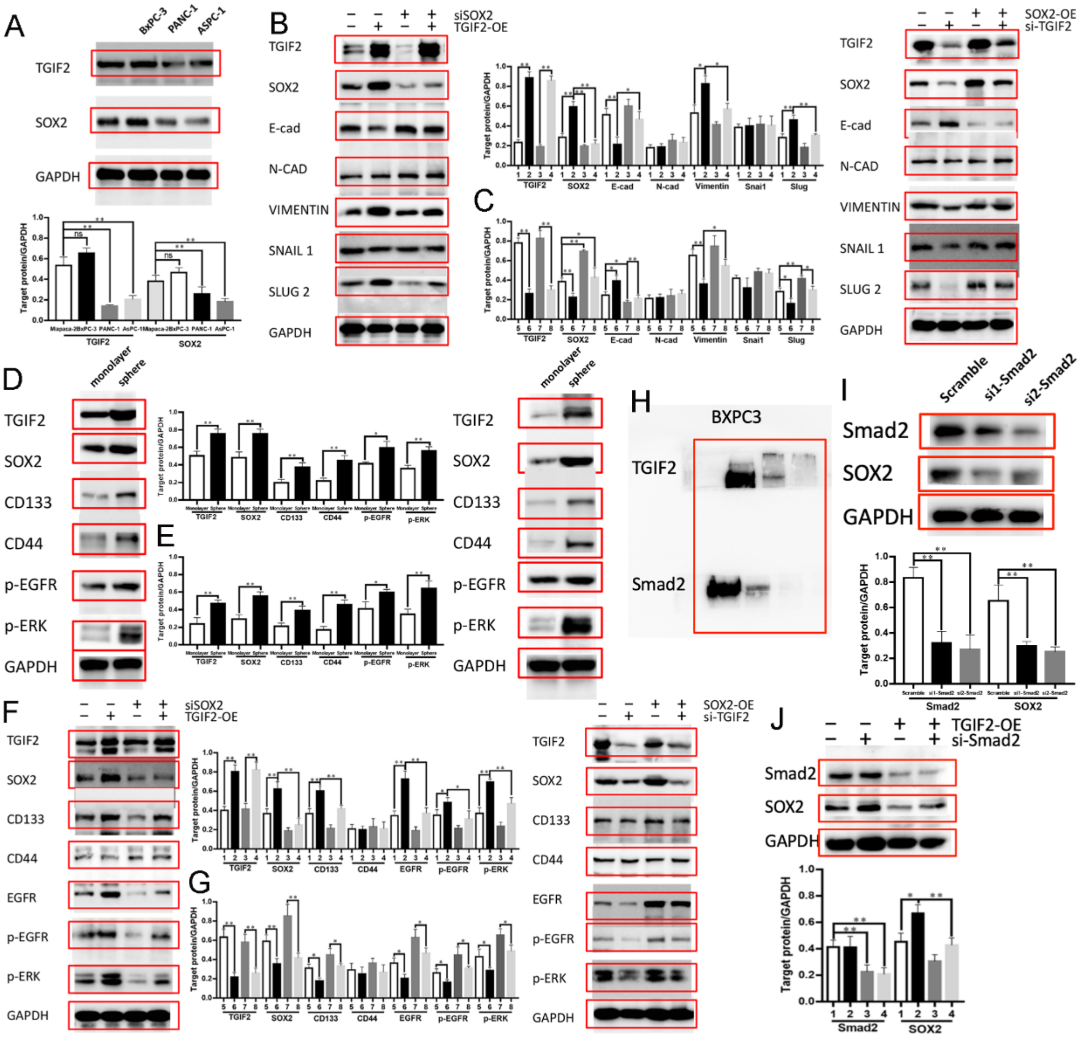

Table S1. Primers used in this study

Primer name	Sequence (5'-3')
TGIF2-F	CGGACAGTGATCTAGGTGAGGACG
TGIF2-R	GGGAAATGGTAAACTGATTAGGGTCTT
SOX2-F	GCTACAGCATGATGCAGGACCA
SOX2-R	TCTGCGAGCTGGTCATGGAGTT
EGFR-F	AACACCCTGGTCTGGAAGTACG
EGFR-R	TCGTTGGACAGCCTTCAAGACC
Slug-F	ATCTGCGGCAAGGCGTTTTCCA
Slug-R	GAGCCCTCAGATTTGACCTGTC
GAPDH-F	CATGAGAAGTATGACAACAGCCT
GAPDH-R	AGTCCTTCCACGATACCAAAGT
SOX2 promotor-F1 (SOX2: -1428 to -1439)	GTGGATGAGCGGGAGAACAA
SOX2 promotor-R1 (SOX2: -1428 to -1439)	GTCACACCACACGCCTTTTC
SOX2 promotor-F2 (SOX2: -1141 to -1132)	GTGGATGAGCGGGAGAACAA
SOX2 promotor-R2 (SOX2: -1141 to -1132)	GTCACACCACACGCCTTTTC
SOX2 promotor-F3 (SOX2: -196 to -187)	GTGGATGAGCGGGAGAACAA
SOX2 promotor-R3 (SOX2: -196 to -187)	GTCACACCACACGCCTTTTC
EGFR promotor-F1 (EGFR:-485 to-495)	AGAGCCGAGAGATCAGGGTT
EGFR promotor-R1 (EGFR:-485 to-495)	CTTGACACAAACAGCCGTGG
EGFR promotor-F2 (EGFR:-705 to -715)	AAAGGCAGGCTGATCGGAAG
EGFR promotor-R2 (EGFR:-705 to -715)	AGAGAATGTCCGGTGGTTCC
Slug promotor-F (Slug: 667 to 677)	GCAGGCTGATCGGAAGAACT
Slug promotor-R	TGTCCGGTGGTTCCAAATGA

(Slug: 667 to 677)	

Table S2. siRNA sequences for TGIF2 and SOX2

Gene	siRNA sequences
si1-TGIF2	CCAGCAGACUCUGACUCAATT
si2-TGIF2	GCACACUCCCAUCCCUUUATT
si1-SOX2	GGACAUGAUCAGCAUGUAUTT
si2-SOX2	CCACCUACAGCAUGUCCUATT
Si-Ctrl	UUCUCCGAACGUGUCACGUTT
sh-TGIF2	CAGGACCCATCACTCCCATTA
sh-SOX2	TTCACATGTCCCAGCACTACC
shRNA Control	TCTCGCTTGGGCGAGAGTAAG

Table S3. The clinicopathological significance of in 20 cases in PC

Parameters	No. of patients
Cases	20
Age(years)	
≤65	12
>65	8
Gender	
Male	14
Female	6
Tumor size(cm)	
<3	12
≥3	8
Tumor location	
Head	16
Body-tail	4
Differentiation	
Well	9
Moderate to Poor	11
T stage [a]	
T1	13
T2+T3	7
LN metastasis [b]	
N0(negative)	17
N1(positive)	3
UICC stage [a]	
I stage	9
II+III stage	11
Vascular permeation	
Absent	12
Present	8

a. According to 8th TNM stage of AJCC. b. Lymph node

Table S4. Common targets of TGIF2 in TFDB 3.0 database.

Target	Position1	Position2
MYC	chr20,36573224,36573541,8.42,-264,pr,dataset-2142	chr20,36574570,36575060,10.3,1082,gb,dataset-2142
SOX2	chr20,36571773,36571869,8.04,-171,pr,dataset-3048	chr20,36571755,36571890,11.7,-173,pr,dataset-3051
TP53	chr20,36573429,36573712,4.94,-59,pr,dataset-3631	chr20,36573429,36573712,4.94,-59,pr,dataset-3631
LMNB1	chr20,36575199,36575676,2.45,1711,gb,dataset-1931	chr20,36565722,36565966,4.68,-776,pr,dataset-1931
YY1	chr20,36573595,36573747,4.62,107,pr,dataset-3761	chr20,36573091,36573470,8.98,-397,pr,dataset-3761
OTX2	chr20,36585545,36585718,4.98,1205,gb,dataset-2384	chr20,36585545,36585718,4.98,1205,gb,dataset-2384
CBX3	chr20,36573062,36573171,2.94,-426,pr,dataset-340	chr20,36574689,36575036,5.31,1201,gb,dataset-340
HDAC1	chr20,36573089,36573819,18.9,-399,pr,dataset-4505	chr20,36573089,36573819,18.9,-399,pr,dataset-4505
